# Sintilimab combined with anlotinib and chemotherapy as second-line or later therapy in extensive-stage small cell lung cancer: a phase II clinical trial

**DOI:** 10.1038/s41392-024-01957-3

**Published:** 2024-09-16

**Authors:** Xiao Han, Jun Guo, Lingyu Li, Yong Huang, Xue Meng, Linlin Wang, Hui Zhu, Xiangjiao Meng, Qian Shao, Xing Li, Yan Zhang, Jin Wang, Yanhua Chen, Yingjie Zhang, Yiru Chen, Changbin Zhu, Zhehai Wang

**Affiliations:** 1 Department of Medical Oncology, Shandong Cancer Hospital and Institute, Shandong First Medical University and Shandong Academy of Medical Sciences, Jinan, Shandong China; 2 Department of Medical Oncology, Shanghe County People’s Hospital, Jinan, Shandong China; 3 Department of Imageology, Shandong Cancer Hospital and Institute, Shandong First Medical University and Shandong Academy of Medical Sciences, Jinan, China; 4 Department of Radiation Oncology, Shandong Cancer Hospital and Institute, Shandong First Medical University and Shandong Academy of Medical Science, Jinan, Shandong China; 5 School of Public Health, Shandong First Medical University & Shandong Academy of Medical Sciences, Jinan, China; 6 Department of Translational Medicine, Amoy Diagnostics, Xiamen, Fujian China

**Keywords:** Lung cancer, Drug development, Tumour biomarkers

## Abstract

Treatment options for patients with relapsed extensive-stage small cell lung cancer (ES-SCLC) remain scarce. This study aims to evaluate the efficacy and safety of combining anlotinib and sintilimab plus chemotherapy as a second line or later therapy for ES-SCLC patients. This is a phase II clinical trial (ChiCTR2100049390) conducting at Shandong Cancer Hospital. Patients with ES-SCLC and received at least one prior systemic treatment were enrolled. The trial design involved a combination therapy (sintilimab, anlotinib, and nab-paclitaxel) administered over six 21-day cycles, followed by maintenance sintilimab therapy. The primary endpoint was objective response rate (ORR). Circulating tumor DNA sequencing was employed for exploratory analysis. From July 2021 to April 2023, 25 eligible patients were enrolled. The confirmed ORR was 60% (95% CI: 38.7–78.9%) and the DCR was 76% (95% CI: 54.9–90.6%). The mPFS was 6.0 months (95% CI: 5.4–9.7), and the 6-month PFS rate was 49.2%. The mOS was 13.4 months (95% CI: 11.8-NR), with a 12-month survival rate of 62.2%. Treatment-related adverse events (TRAEs) of any grade occurred in 80% of patients, with the most common being fatigue (40%) and nausea (32%). TRAEs of Grade 3 or higher were reported in 12% of patients. ctDNA analysis indicated that low on-treatment blood tumor mutation burden was associated with longer PFS and OS and a potential role of *KMT2D* mutation in treatment resistance. This combination therapy shows promising efficacy and a manageable safety profile as a second-line or later treatment for ES-SCLC, with genomic insights providing potential biomarkers for treatment response.

## Introduction

Extensive-stage small cell lung cancer (ES-SCLC) is characterized by a dismal three-year survival rate of merely 6% and continues to present a formidable challenge in oncology.^1^ Platinum-based chemotherapy remains the mainstay of current first-line standard care and may be optionally combined with immune checkpoint inhibitors like atezolizumab or durvalumab. Adding immunotherapy to chemotherapy has been shown to modestly increase median overall survival (mOS) by approximately 2–3 months, reducing the risk of death by about 30%.^2^ Despite these advances, disease progression is almost inevitable, leading to a dire need for efficacious treatment options for patients with relapsed disease.

The landscape of second-line treatments for ES-SCLC has, however, been relatively stagnant. Topotecan, approved in 1996, remains the most commonly used agent globally despite its limited efficacy and unfavorable safety profile.^3^ In a significant development, lurbinectedin received FDA approval in 2020 based on its reported 35% objective response rate (ORR) in a second-line setting.^4^ However, this promising antitumor activity did not translate into improved overall survival in the subsequent phase 3 ATLANTIS trial. The third-line treatment landscape is even more bleak, with no specific FDA-approved agents for relapsed SCLC following the withdrawal of approvals for nivolumab and pembrolizumab in 2020 and 2021, respectively.^5^ In this context, antiangiogenic therapy, alone or in combination with immunotherapy, emerged as a potential avenue for treatment development in relapsed SCLC, albeit with mixed efficacy results. Antiangiogenic therapies including agents like bevacizumab, sorafenib, sunitinib, and surufatinib have shown limited efficacy, with the notable exception of the multitargeted tyrosine kinase (TK) inhibitor anlotinib.^6–9^ In the ALTER 1202 study, third-line anlotinib treatment significantly extended median PFS by 3.4 months in ES-SCLC, alongside improvements in mOS and disease control rate (DCR).^10^ This led to a National Medical Products Administration approval in 2019 for ES-SCLC in China in third-line settings or beyond.

Currently, anlotinib in combination with immune checkpoint inhibitors (ICIs), such as sintilimab, has shown promising results in solid malignancies, with existing findings pointing toward encouraging efficacy and good safety profile.^11–13^ Nab-paclitaxel has been shown to exert a synergistic effect with immunotherapy agents,^14,15^ potentially enhancing treatment outcomes. Evidence supports nab-paclitaxel with modest antitumor activity and favorable toxicity profile in relapsed SCLC.^16^ Nevertheless, immunotherapy combined with TKIs plus chemotherapy has not yet been explored in relapsed SCLC patients, despite the potential for more potent synergistic effects to enhance treatment efficacy. Nab-paclitaxel obviates the need for premedication with corticosteroids, aligning well with the current ambiguity regarding the impact of corticosteroids on patients undergoing immunotherapy. Although there is no conclusive evidence to suggest that corticosteroids reduce the efficacy of immunotherapy,^17^ both corticosteroids and immunotherapy modulate the immune system through distinct mechanisms. Thus, nab-paclitaxel was chosen as the chemotherapy agent in this investigation, specifically to circumvent the requirement for corticosteroid premedication. This deliberate decision in the study design was aimed at removing this potential confounding influence.

This phase II clinical trial therefore aimed to evaluate the efficacy and safety of sintilimab in combination with anlotinib and chemotherapy as the second-line or later therapy in relapsed SCLC patients.

## Results

### Patient enrollment and baseline characteristics

**Fig. 1:**
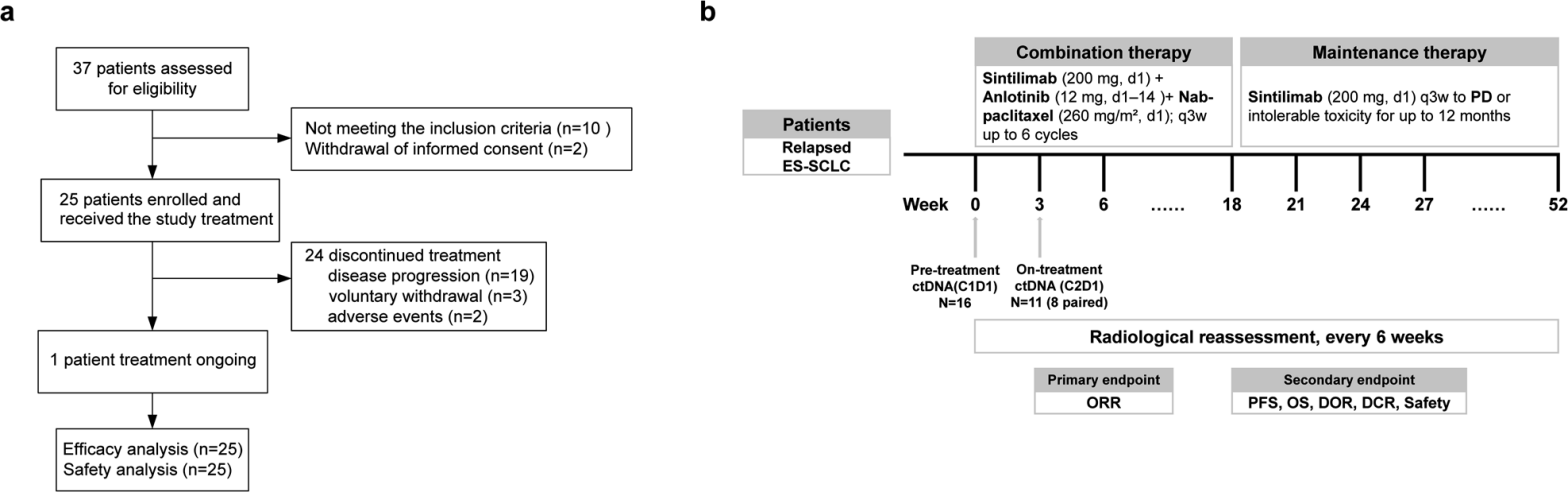
Participant flow diagram and trial plan. **a** Participant flow diagram. **b** Trial plan

Between July 15, 2021, and April 4, 2023, a total of 37 patients were evaluated for eligibility. Of these, 10 were excluded for not meeting the inclusion criteria, and 2 withdrew their informed consent. Consequently, 25 patients were deemed eligible and subsequently enrolled. All enrolled patients received at least one dose of the study drug. Baseline characteristics of the 25 subjects are shown in Table 1. The median age of the cohort was 59 (range: 44–76) years. The majority of patients were male (80%, *n* = 20), under the age of 65 (84%, *n* = 21), and had a grade 1 ECOG performance status (84%, *n* = 21). A slight majority were never-smokers (52%, *n* = 13). At initial diagnosis, 60% (*n* = 15) of the patients had limited-stage SCLC, while 40% (*n* = 10) had extensive disease. Brain metastasis was present in 40% (*n* = 10) of the patients. All participants had undergone prior systemic treatment, with 60% (*n* = 15) having received first-line treatment only and the remaining 40% (*n* = 10) having also received second-line therapies. As of September 30, 2023, 24/25 participants had discontinued the treatment in the study for various reasons: disease progression (*n* = 19), voluntary withdrawal (*n* = 3), and adverse events (*n* = 2) (Fig. 1a). Treatment was ongoing for the remaining patient.

**Table 1:** Baseline characteristic of patients

Characteristic	*N* = 25
Age, median (range), years	59 (44-76)
Age, *n* (%)	
<65 years	21 (84.0)
≥65 years	4 (16.0)
Sex, male, *n* (%)	20 (80.0)
ECOG PS, *n* (%)	
1	21 (84.0)
2	4 (16.0)
Smoking status, *n* (%)	
Never	13 (52.0)
Current/Former	12 (48.0)
Disease classification at initial diagnosis, n (%)	
Extensive disease	10 (40.0)
Limited disease	15 (60.0)
Previous systemic therapies, *n* (%)	
First-line	15 (60.0)
Second-line	10 (40.0)
Brain metastasis, *n* (%)	
Yes	10 (40.0)
No	15 (60.0)
Prior platinum-based therapy	
Yes	25 (100.0)
No	0

*ECOG PS* Eastern Cooperative Oncology Group Performance Status

### Efficacy results

**Fig. 2:**
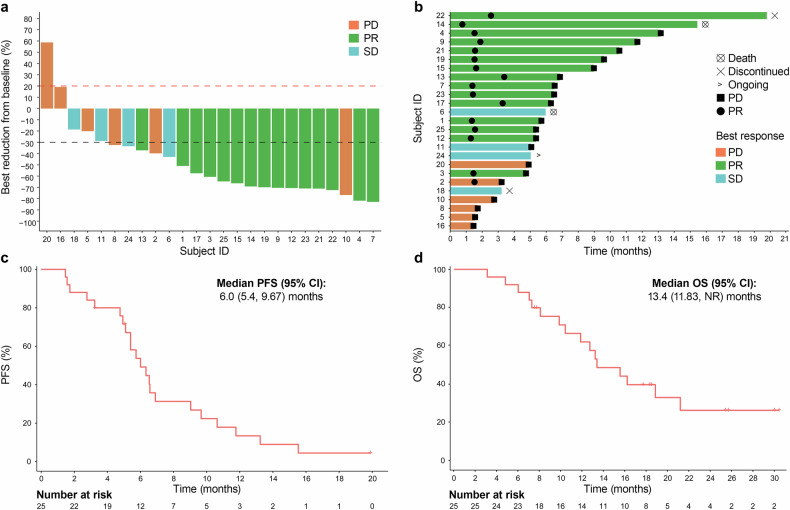
Anti-tumor activity and survival outcomes. **a** Best percentage of change from baseline in the sum of the diameters of target lesions. Dashed lines at +20% and −30% delineate the thresholds for PD and PR, respectively, following RECIST v1.1 criteria. **b** Duration of each patient’s response to treatment. **c** Kaplan–Meier curve for PFS. **d** Kaplan–Meier curve for OS. Note: Median PFS and OS are indicated in **c** and **d**

The median duration of follow-up was 13.0 (range: 3.0–30) months. Among the 25 patients, 15 exhibited a confirmed partial response (PR) and 4 had stable disease (SD) (Table 2 and Fig. 2a). The confirmed ORR was 60% (95% CI: 38.7–78.9%) and the DCR was 76% (95% CI: 54.9–90.6%). The median time to response was 1.9 (95% CI: 1.57–NA) months. The median DOR was 5.2 (95% CI: 4.1–11.7) months and the longest was 19.3 months (Fig. 2b). In 14 patients (58.3%), deep response was seen with 50% reduction at post-baseline assessment (Fig. 2a). Median PFS (mPFS) was 6.0 (95% CI: 5.4–9.7) months (Fig. 2c), with a 6-month PFS rate of 49.2% (Table 2). The mOS was 13.4 (95% CI: 11.8–NR) months (Fig. 2d) and the 12-month OS rate was 62.2% (Table 2).

**Table 2:** Anti-tumor activity

Variables	*n* = 25
Best objective response *n* (%)	
Complete response (CR)	0
Partial response (PR)	15 (60.0)
Stable disease (SD)	4 (12.0)
Progressive disease (PD)	6 (35.0)
ORR *n* (%; 95% CI)	15 (60.0; 49.4–78.6%)
DCR *n*, (%; 95% CI)	19 (76.0; 62.4–89.6%)
TTR (months)	1.48 (95% CI: 1.42–1.54)
DOR (months)	4.99 (95% CI: 4.18–7.69)
mPFS (months) 6-month PFS rate	6.41 (95% CI: 4.8–8.9) 56.0% (95% CI: 34.79–72.73)
mOS	13.3 (95% CI: 9.8–21.1)
12-month OS rate	62.2% (95% CI: 39.77–78.34)

### Safety results

All 25 patients were administered at least one dose of the drugs under study and were evaluated for safety. Therefore, the safety analysis included all 25 participants. Median duration of treatment was 6.0 (95% CI: 4.1–7.9) months. Twenty-three (92%) patients experienced at least one TRAE of any grade, with the most common being leukopenia (*n* = 14, 56.0%), anaemia (*n* = 13, 52.0%), and elevated γ-glutamyltransferase (GGT) (*n* = 12, 48.0%) (Table 3). TRAEs of grade 3 or 4 occurred in 4 patients (16%), with elevated aspartate aminotransferase (AST) (*n* = 3, 12%) being the most common. Serious adverse events occurred in three patients: grade 3 elevated AST (*n* = 2, 8%) and grade 3 rash (*n* = 1, 4%). TRAEs leading to drug discontinuation occurred in two patients (8%). No treatment-related deaths occurred. Eleven patients (44%) experienced immune-related adverse events, with hypertriglyceridemia (*n* = 4, 16%) being the most common.

**Table 3:** Summary of TRAEs

TRAEs	All grade (*n* = 25)	Grade ≥ 3(*n* = 25)
Leukopenia	14 (56)	1 (3.6)
Anaemia	13 (46.4)	0
Elevated GGT	12 (42.9)	0
Elevated direct bilirubin	11 (39.3)	0
Leukopenia	10 (35.7)	0
Decreased hemoglobin	10 (35.7)	0
Asthenia	7 (25.0)	0
Elevated AST	7 (25.0)	2 (7.1)
Thrombocytopenia	7 (25.0)	0
Peripheral sensory neuropathy	7 (25.0)	0
Neutropenia	6 (21.4)	1 (3.6)
myelosuppression	5 (17.9)	0
Hypertriglyceridemia	4 (14.3)	0
Elevated indirect bilirubin	4 (14.3)	0
Drug-induced liver injury	4 (14.3)	0
Decreased appetite	3 (10.7)	0

The most common (≥10% participants) anlotinib-related AEs were fatigue and peripheral neurotoxicity, which were reported in 7 (28%) and 5 (20%) participants, respectively. The incidence of other anlotinib-related AEs, including proteinuria and diarrhea, was low (reported in single [4%] participant). The most common sintilimab-related AEs was hypertriglyceridemia, which was reported in 4 (16.0%) participants. Other sintilimab-related AEs, including but not limited to elevation in liver enzymes ALT and AST, thyroid-stimulating hormone, and free thyroxine, were reported in no more than 2 (8.0%) participants.

### Exploratory analysis of ctDNA in SCLC patients

**Fig. 3:**
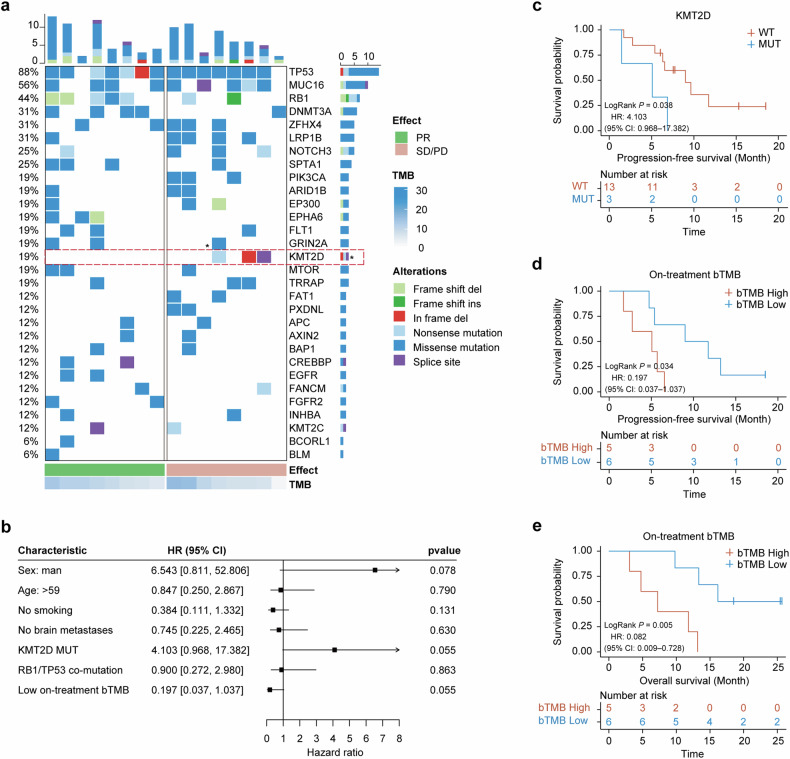
Genomic insights and survival analysis based on ctDNA profiling. **a** Heatmap showing the distribution and frequency of key genetic mutations across the study cohort. **b** Forest plot showing HR results for the patient characteristics indicated. **c** Kaplan–Meier plot highlighting the differential impact of *KMT2D* mutations on PFS, with a statistically significant decrease in PFS for MUT patients. **d, e** Survival analysis comparing PFS and OS in patients with high versus low on-treatment bTMB, illustrating the prognostic value of bTMB

Baseline and post-treatment ctDNA profiles of patients with relapsed SCLC were comprehensively analyzed to explore their associations with treatment responses and survival outcomes (Fig. 1b). Twenty-seven peripheral blood samples were utilized for this analysis, including 16 samples collected during the pretreatment period and an additional 11 samples obtained after the first treatment cycle. The results reveal that the most frequently mutated genes included *TP53* (88%), *MUC16*(56%), *RB1*(44%), *DNMT3A* (31%), *ZFHX4* (31%) (Fig. 3a). These mutations are consistent with previously reported profiles in SCLC, indicating a typical mutation spectrum for this patient cohort.^11,18^

Using a forest plot we summarized the hazard ratios (HRs) for clinical and genetic characteristics and their impact on patient outcomes (Fig. 3b). Notably, the presence of KMT2D mutations showed a trend towards a negative impact on survival, with an HR of 4.103 (95% CI: 0.968–17.382, *p* = 0.055). The Kaplan–Meier curve also illustrates patients with KMT2D mutations experienced shorter PFS (Log-Rank *p* = 0.038) (Fig. 3c). These results indicated a potential role of KMT2D mutations in treatment resistance.

Further analysis of bTMB variations in relation to treatment responses explored the connection between genetic changes and clinical outcomes in more detail. The difference between pretreatment and on-treatment bTMB, expressed as ΔbTMB, proved greater for patients who achieved PR than for those with stable or progressive disease (SD/PD), and while this did not reach statistical significance (*p* = 0.11), a noticeable trend was evident (Supplementary Fig 1). In parallel, low on-treatment bTMB was correlated with improved outcomes, with an HR of 0.197 (95% CI: 0.037–1.037, *p* = 0.055) (Fig. 3b). In addition, patients exhibiting high on-treatment bTMB levels during treatment demonstrated markedly improved PFS (Log-rank *P* = 0.034) and OS (Log-rank *P* = 0.005) (Fig. 3d, e). These data suggested on-treatment bTMB as a predictive biomarker for therapeutic efficacy.

### Functional and epigenetic consequences of *KMT2D* mutations

**Fig. 4:**
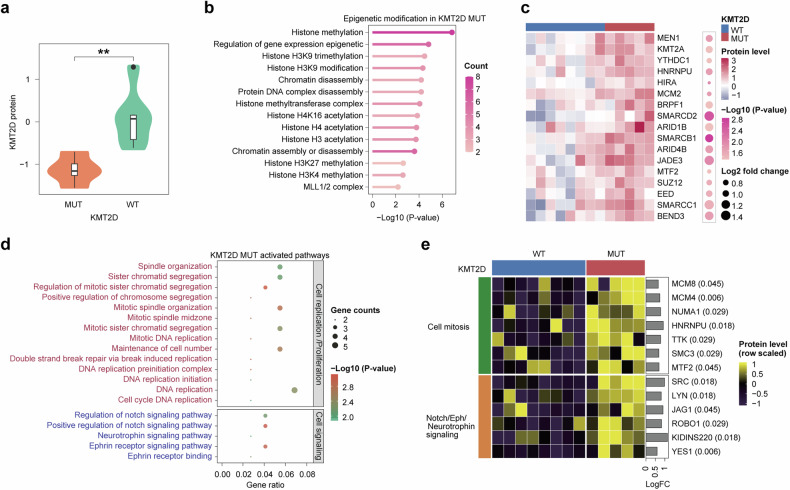
Impact of *KMT2D* mutations on protein expression and cellular pathways. **a** Violin plots comparing KMT2D protein levels between MUT and WT subgroups. The data indicate significantly lower protein expression in MUT patients, suggesting an impact of mutant *KMT2D* on protein stability or production (*p* < 0.01). **b** Bubble chart illustrating the epigenetic modifications predominantly associated with *KMT2D* mutations. Significant pathways affected include histone methylation and chromatin assembly, with the strongest changes observed in histone H3K9 trimethylation, suggesting an influence of *KMT2D* mutations on gene silencing. **c** Heatmap of protein expression profiles contrasting MUT and WT patients. Key regulatory proteins such as MEN1 and SUZ12 exhibit differential expression; this may contribute to altered cellular functions and cancer progression in MUT patients, potentially underlying apparent treatment resistance. **d** Plot showing the pathways activated in *KMT2D* MUT versus WT cells. Enhanced activities in pathways related to cell cycle regulation, DNA replication, and chromosomal segregation are evident, emphasizing the oncogenic potential of mutant *KMT2D*. **e** Heatmap comparing the activity of various cellular pathways between *KMT2D* MUT and WT cell lines. This visualization emphasizes the differential regulation, evidenced through protein expression changes, of key pathways such as cell mitosis and Notch/ephrin/neurotrophin signaling, showcasing how *KMT2D* mutations may affect cellular functions and influence treatment outcomes

In view of the above results, we conducted a comparative analysis of KMT2D protein levels between the MUT and WT subgroups, revealing a significant reduction in the MUT group (Fig. 4a). The violin plot shows notably decreased KMT2D expression in the presence of mutations (*p* < 0.01). We also identified significant epigenetic changes associated with *KMT2D* mutation (Fig. 4b), highlighting the regulatory impact of such mutation on gene expressions. Among others, the key affected pathways included histone methylation, chromatin disassembly, and protein–DNA complex disassembly, reflecting the known roles of the KMT2D protein. Enrichment analysis indicated that histone H3K9 trimethylation, a critical marker of gene silencing, was the most significantly impacted process (*p* < 0.01), underscoring the profound epigenetic influence exerted by KMT2D mutations in SCLC.

A heatmap of differential protein expression profiling results between KMT2D MUT and WT samples is presented in Fig. 4c, demonstrating that several key regulatory proteins—including MEN1(log2FC = 0.944, *p* = 0.002) and SUZ12(log2FC = 0.781, *p* = 0.003), among others—showed considerable variation in expression levels, thereby suggesting a broad impact of KMT2D mutations beyond simple genetic disruption. In the heatmap, color intensity is used to indicate the degree of change in expression, with significant changes validated through statistical analysis.

The pathways most significantly activated in KMT2D mutant versus WT cells were also identified. Enhanced activity was found in pathways related to cell cycle regulation, DNA replication, and chromosomal segregation (Fig. 4d), suggesting that KMT2D mutations may contribute to oncogenesis through disruption of normal cell cycle and mitotic processes. The heatmap in Fig. 4e displays a number of proteins with statistically significant variation in cells based on *KMT2D* mutation status. Proteins involved in cell mitosis (such as NUMA1, SMG6, and TTK) and Notch/ephrin/neurotrophin signaling (including JAG1 and ROBO1) showed significant upregulation in the presence of mutant *KMT2D*. This may be useful to guide targeted therapeutic strategies in clinical settings.

### Modulation of cellular and immune pathways by *KMT2D* mutations

**Fig. 5:**
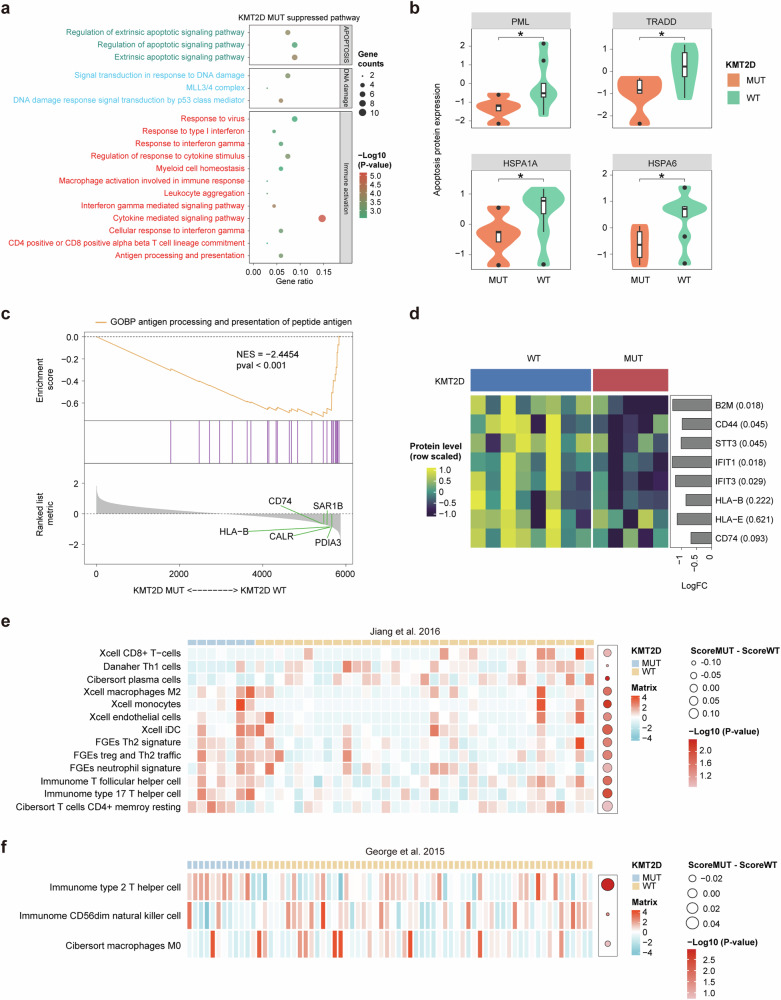
Comprehensive analysis of *KMT2D* mutation effects on immune and cellular pathways. **a** Bubble plot displaying the pathways significantly suppressed in *KMT2D* MUT cells, highlighting disruptions in apoptotic signaling and immune response pathways. This visualization summarizes the extensive impact of mutant *KMT2D* on cellular communication and apoptosis regulation. **b** Violin plots illustrating altered protein expression levels in apoptosis-related proteins such as PML, TRADD, HSPA1A, and HSPB6 between the MUT and WT groups. Decreased levels of these critical proteins are found in MUT samples, suggesting a role for *KMT2D* mutations in apoptosis evasion. **c** GSEA shows negative enrichment in antigen processing and presentation pathways in MUT samples, with notable downregulation of key proteins involved in antigen presentation, such as CD74 and SAR1B, highlighting potential immune evasion by mutant cells. **d** Heatmap depicting expression of immune-modulatory proteins across both groups, illustrating distinctive expression patterns that may affect the immune surveillance capabilities of the tumor microenvironment in MUT patients. **e** Heatmap showing profiles across various tumor immune cell types, including CD8^+^ T cells, Th1 cells, and M2 macrophages. This shows the influence of mutant *KMT2D* on the immune cell landscape within the tumor microenvironment, potentially impacting tumor progression and therapy response. **f** Heatmap comparing the differential presence of specific immune cell types, such as Th2 cells and NK cells. This analysis offers insights into how *KMT2D* mutations might lead to diverse tumor–immune system interactions and affect patient outcomes

We also found significant pathway suppression associated with *KMT2D* mutations, particularly in apoptotic signaling and immune response pathways (Fig. 5a). Furthermore, comparative protein expression analysis between the MUT and WT subgroups (Fig. 5b) demonstrated significant downregulation of key apoptosis-related proteins such as PML, TRADD, HSPA1A, and HSPB6 in the presence of *KMT2D* mutations, indicating a broad impact on cellular stress responses and apoptotic mechanisms.

Moreover, GSEA highlighted notable downregulation of antigen processing and presentation pathways including key proteins such as CD74 and SAR1B, with a significant negative enrichment score (NES = −2.4454, *p* < 0.001; Fig. 5c), highlighting potential immune evasion by mutant cells in *KMT2D* MUT samples. This is further underscored by the heatmap (Fig. 5d) detailing expression levels of immune-modulatory proteins between WT and MUT groups, showing distinct expression patterns for proteins such as B2M, STAT3, and various HLA molecules, which are crucial for immune surveillance and response. This differential protein expression profile suggests a mutation-induced alteration in the immune evasion tactics of tumor cells and may explain the phenotypic association of *KMT2D* mutations with treatment nonresponse.

Further exploratory analysis using gene expression profiles (Fig. 5e) of SCLC patients indicated that *KMT2D* mutations affect various immune cell populations within the tumor microenvironment, including CD8^+^ T cells, Th1 cells, and M2 macrophages. We extended our analysis to the impact of *KMT2D* mutations on the tumor microenvironment with another patient cohort (Fig. 5f), finding variations in the presence of specific immune cell types such as T helper 2 (Th2) cells and natural killer (NK) cells, and providing insights into how genetic mutations can lead to heterogeneous tumor–immune interactions across different patient populations.

## Discussion

To the best of our knowledge, this is the first clinical study reporting integration of anlotinib plus sintilimab treatment with nab-paclitaxel in relapsed SCLC patients. Our findings indicate promising efficacy for this therapeutic regimen in second-line or later settings. The confirmed ORR in the present trial was 60% (95% CI: 38.7–78.9%) and the DCR was 76% (95% CI: 54.9–90.6%). The mPFS was 6.0 (95% CI: 5.4–9.7) months, and the 6-month PFS rate was 49.2%. The mOS was 13.4 (95% CI: 11.8–NR) months, with a 12-month survival rate of 62.2%. The primary endpoint was reached in this study. In addition, this study makes a significant contribution to the limited existing prospective research on ctDNA characteristics and dynamics in ES-SCLC patients following therapeutic intervention.

Emerging therapies for relapsed SCLC patients have typically achieved an ORR of less than 50% and an mPFS of under six months.^19^ Treatment of these patients with carboplatin and nab-paclitaxel has shown limited effectiveness, achieving an ORR of 19.0% (95% CI: 6.8–38.4%), an mPFS of 2.5 (95% CI: 1.5–3.4) months, and an mOS of 5.1 (95% CI: 2.1–8.1) months.^20^ A phase II study of talazoparib with temozolomide demonstrated the effectiveness of integrating a PARP inhibitor with chemotherapy, reporting a 39.3% ORR alongside an mPFS of 4.5 months.^21^ Additionally, in an investigation of tarlatamab (an anti-DLL3 antibody) for relapsed SCLC, the ORR was 23.4% (95% CI: 15.7–32.5) and the mPFS was 3.7 (95% CI: 2.1–5.4) months.^22^ Another trial compared berzosertib plus topotecan against topotecan alone for relapsed SCLC, finding an mPFS of 3.9 vs. 3.0 months, while the mOS was 8.9 vs. 5.4 months.^23^ Elsewhere, a phase III study in relapsed patients found that combined dinutuximab and irinotecan therapy did not improve outcomes or response rates compared to irinotecan or topotecan alone: mOS was 6.9 vs. 7.0 vs. 7.4 months (*p* = 0.3132); mPFS was 3.5 vs. 3.0 vs. 3.4 months (*p* = 0.3482); ORR was 17.1% vs. 18.9% vs. 20.2% (*p* = 0.8043); and the clinical benefit response (CBR) rate was 67.4% vs. 58.9% vs. 68.1% (*p* = 0.0989) for the three treatment arms, respectively.^24^

The promising efficacy observed in this study may result from synergistic effects of the three combination drugs. Anlotinib has a role in altering the tumor microenvironment to make it more conducive to immune therapy, and its potential to improve drug delivery via vascular normalization is significant.^25,26^ This agent has also been observed to augment the infiltration of innate immune cells, in turn enhancing the efficacy of PD-1 blockade.^25^ It additionally possesses the capability to attenuate PD-L1 expression via deactivation of the AKT pathway, thereby facilitating remodeling of the tumor microenvironment as mentioned above.^27^ Moreover, synergetic effects of the same drugs have been observed in several advanced solid malignancies. A prospective, single-arm, phase II trial investigated this combination in NSCLC patients harboring uncommon EGFR mutations, demonstrating an mPFS of 7 months along with 20-month mOS.^28^ Another phase II trial assessed the same combination in PD-L1-positive recurrent/metastatic cervical cancer, reporting a 54.8% ORR with an mPFS of 9.4 months.^13^ In the context of SCLC, a phase II trial involving camrelizumab combined with apatinib in relapsed patients found a 34.0% ORR, an mPFS of 3.6 months, and an mOS of 8.4 months.^29^ While a retrospective study in China suggested that anlotinib plus immunochemotherapy results in an mPFS of 5.7 months for relapsed ES-SCLC,^30^ our study represents the inaugural prospective clinical trial confirming promising efficacy when combining anti-PD-1 antibodies with antiangiogenic agents in patients with ES-SCLC.

The integration of nab-paclitaxel into the treatment regimen achieved a high confirmed ORR of 60% in our study. Under certain conditions, chemotherapeutic agents have been suggested to display immune-enhancing effects and to augment CD8^+^ T-cell-dependent immune surveillance and rejection of cancer cells.^31^ Specifically, chemotherapy modulates the immune environment by reducing immunosuppressive cell activities, reviving antigen-specific CD8^+^ T cells, normalizing tumor neovasculature to promote CD8^+^ T cell infiltration, and boosting major histocompatibility complex I expression and neoantigen presentation in tumor cells. Additionally, it induces immunogenic tumor cell death and augments tumor cell sensitivity to IFN-γ. These mechanisms highlight the dual benefits of this treatment strategy: direct antitumor effects and immune system enhancement. The potential of this therapeutic approach is further supported by ongoing clinical trials exploring similar combination strategies, such as the ICI benmelstobart plus anlotinib/chemotherapy for ES-SCLC. Additionally, trials involving combinations like carboplatin, etoposide, bevacizumab, and atezolizumab underscore a growing trend toward leveraging this multifaceted strategy in the treatment of ES-SCLC.

To date, monitoring of ctDNA has not been sufficiently developed for routine use in ES-SCLC.^32^ Our study adds valuable data in this regard, noting that the spectrum of ctDNA alterations we detected in ctDNA reflects current knowledge on the genomic landscape of SCLC. Low on-treatment blood tumor mutation burden (bTMB) was associated with improved clinical outcomes. We identify mutations in the *KMT2D* gene as significant potential contributors to therapy resistance. Other study suggested a predictive value of the *KMT2D* gene in NSCLC,^33^ since its deficiency leads to enhanced tumorigenesis. Loss of *KMT2D* impacts glycolysis pathways, making lung cancer cells more vulnerable to glycolytic inhibitors,^34^ and also leads to increased activity of receptor TKs such as EGFR and ERBB2/HER2.^35^ By contrast, our analysis highlights extensive epigenetic modifications associated with KMT2D mutations, particularly affecting histone methylation at H3K4, H3K9 and H3K27. These changes are critical for chromatin dynamics and gene expression regulation.^36^ Such epigenetic alterations could potentially modulate the tumor microenvironment to favor immune escape, thereby reducing the efficacy of immunotherapeutic agents such as sintilimab. Moreover, downregulation of key antigen presentation pathway genes and reduced expression of several immune-modulatory proteins accompanying *KMT2D* mutations were identified in this study may strengthen the effects. Our analysis highlights significant changes in immune cell distributions within the tumor microenvironment, marked by reduced pro-inflammatory cell infiltration and increased presence of Th2 cells. The altered tumor-immune interactions, immune evasion, and reduced apoptosis induced by KMT2D mutations were possibly precipitating resistance to treatment. Our findings also reveal upregulation of the NOTCH/Eph and neurotrophin pathways in cells harboring KMT2D mutations, identifying novel potential targets for therapeutic intervention.

The insights gained through this study, while illuminating the potential benefits of sintilimab combined with anlotinib and chemotherapy for ES-SCLC, are constrained by several limitations. First, the relatively small number of participants restricts the breadth of our conclusions and dampens the comparative strength of our evidence. Second, executing a single-center study narrows the ethnic and genetic variance of the participant pool, which may curtail the broader applicability of our results. Moreover, the exploratory evaluations conducted on ctDNA, particularly regarding the implications of *KMT2D* mutations, must be interpreted prudently. Due to the nature of this study, it was not possible to obtain sufficient biospecimens from late-stage SCLC patients for detailed transcriptomic and proteomic analysis, leading us to instead rely on publicly available datasets. Nevertheless, our findings pave the way for further investigations to inform targeted therapies, and randomized phase III clinical trials are warranted to compare the present regimen with topotecan or amrubicin in relapsed ES-SCLC.

In conclusion, this study represents a significant advancement in the treatment of relapsed ES-SCLC, offering crucial insights into ctDNA-based monitoring and the identification of potential biomarkers for resistance to this combination therapy. These findings contribute transformative insights into the treatment landscape for relapsed SCLC, highlighting a therapeutic strategy that holds substantial promise for enhancing patient outcomes.

## Material and methods

### Subjects and trial design

This phase II clinical trial was carried out at Shandong Cancer Hospital, China (ChiCTR2100049390). The primary objective was to assess the efficacy and safety of the combination of anlotinib plus sintilimab with chemotherapy as a second-line or later treatment in ES-SCLC. The research adhered to the principles of the Declaration of Helsinki and the Good Clinical Practice (GCP) guidelines. For the study protocol, approval was obtained from the institutional review board and ethics committee of Shandong Cancer Hospital (SDZLEC2021-055-02). Prior to participation, written informed consent was provided by all patients. The study was additionally endorsed by the China Center for Drug Evaluation (CDE) of the National Medical Products Administration (NMPA).

The inclusion criteria were patients aged 18–75 years with cytologically or histologically confirmed SCLC according to the Veterans Administration Lung Study Group (VALG) staging system; one or more measurable lesions as defined by the Response Evaluation Criteria in Solid Tumors version 1.1 (RECIST v1.1); a minimum of one prior systemic treatment for SCLC in the metastatic setting; a grade 0 or 1 performance status on the Eastern Cooperative Oncology Group (ECOG) scale; adequate bone marrow and organ function; and clinically and radiologically stable brain metastases (if present) following treatment. The main exclusion criteria were defined as patients with prior anti-PD-1/L1/L2 antibody treatment, prior therapies targeting stimulatory or coinhibitory T-cell receptors (e.g., CD137, CTLA-4, OX-40), life expectancy of less than three months and patients with specific pretreatment conditions (including the use of glucocorticoids within seven days prior to enrollment). A washout period was mandated depending on the nature of previous treatments. For detailed inclusion and exclusion criteria, please refer to the full study documentation available at chinadrugtrials.org.cn.

### Interventions

The intervention protocol involved a combination therapy administered over a maximum of six 21-day cycles. Each cycle consisted of sintilimab (200 mg intravenously [i.v.] on day 1), anlotinib (12 mg orally [p.o.] on days 1–14), and albumin-bound (nab®) paclitaxel (administered intravenously at 260 mg/m^2^ on day 1). Following these cycles, sintilimab was continued as a maintenance therapy at a dose of 200 mg intravenously every three weeks for up to 12 months. The continuation of treatment was contingent on the absence of disease progression, intolerable side effects, or patient consent being withdrawn. During the study period, participants were not permitted to receive any other antitumor therapy until confirmed disease progression. Where intolerable adverse events led to the delay or discontinuation of one of the combination drugs, affected patients were permitted to receive the remaining drugs and continue participating in the study.

### Endpoints and assessments

The primary endpoint in this study was ORR. Secondary endpoints included progression-free survival (PFS), overall survival (OS), duration of response (DOR), and disease control rate (DCR), as well as safety. Specifically, PFS was taken as the time of the first dose of any drug to that of radiologically assessed progressive disease or death; OS was measured from the first dose of any drug to the time of death, regardless of cause. Tumor responses were assessed every six weeks (±7 days) starting from day one of cycle one, following RECIST v1.1 criteria. Confirmatory scans for complete or partial response were scheduled for at least four weeks after the initial response. Patients showing initial radiologic evidence of progressive disease were allowed to continue treatment until confirmation of progression on subsequent scans if they were considered as benefiting from the ongoing treatment by the investigator. The U.S. National Cancer Institute’s Common Terminology Criteria for Adverse Events, version 5.0 (CTCAE v5.0) framework was used to categorize adverse events.

### Sequencing and analysis of circulating tumor DNA (ctDNA)

Serial blood samples were scheduled to be collected from each study participant before treatment administration on cycle 1, day 1 (C1D1, pre-treatment) and cycle 2, day 1 (C2D1, 3 weeks). Blood samples underwent plasma separation and ctDNA extraction via the QIAamp Circulating Nucleic Acid Kit (Qiagen, Germany). Peripheral blood lymphocyte DNA was extracted with the AmoyDx Blood and Leukocyte DNA Kit (AmoyDx, China) and used as a control. We conducted targeted NGS on these samples using a 571-gene cancer panel (AmoyDx, China) on the Illumina NovaSeq 6000, targeting a depth range of 5600 to 17,500 times. Sequencing outputs, saved as FASTQ files, were processed with ADXMaster-DNA_v0.4.3 (AmoyDx, China). After adapter trimming and quality filtering, the data were aligned to hg19 using BWA-MEM. Duplicate removal was performed using FormatFastq (AmoyDx, China), with ctDNA duplicates identified via SSBC. Variant calling for single nucleotide variants (SNVs) and indels was executed using SSBC-VarScan and IndelCaller, respectively, and annotations were added with Annotator (AmoyDx, China). We classified ctDNA as positive (≥2 mutations) or negative (≤1 mutation) based on the detectability of somatic mutations; the dynamics of the maximum somatic allele mutation frequency (MSAF) were also tracked at baseline and post-treatment. Three criteria for competent mutations were applied: (i) somatic mutations only (not germline), (ii) mutations located in coding sequences and (iii) nonsynonymous SNVs/indels. Then, blood-based tumor mutational burden (bTMB) was computed as the number of competent mutations divided by the length of the genomic region covered by the gene panel.

### Data and resources

To further investigate the impact of KMT2D mutations on downstream signaling pathways, we utilized data from several public databases. Genomic and proteomic expression data for SCLC were downloaded from the Cancer Cell Line Encyclopedia (CCLE) (https://portals.broadinstitute.org/ccle).^37^ Additionally, we incorporated genomic and transcriptomic data from two distinct populations: a Chinese cohort (Gene Expression Omnibus GEO database, GSE60052),^38^ and a Caucasian cohort (cBioPortal, Dataset ID: EGAD00001001244).^18^ These datasets were specifically selected to examine the influence of KMT2D mutations on the tumor immune microenvironment.

### Differential protein expression and tumor immune microenvironment analysis

Differential protein expression between KMT2D mutant and wild-type samples was analyzed using the wilcox.test(stats4_4.0.3). The differentially expressed proteins were then subjected to Gene Ontology (GO) and Kyoto Encyclopedia of Genes and Genomes (KEGG) enrichment analyses. Gene Set Enrichment Analysis (GSEA) was performed using the clusterProfiler package (v4.4.4) in R, with an adjusted *p*-value (<0.05) considered statistically significant. Gene Set Variation Analysis (GSVA) was conducted to score several tumor microenvironment (TME) features, including functional gene expression signatures (FGEs),^39^ Immunome,^40^ and Danaher.^41^ CIBERSORT.^42^ and xCell.^43^ analyses were performed using the R packages CIBERSORT (IOBR v0.99.9) and xCell (v1.1.0), respectively.

### Statistical analysis

In this single-arm, phase II trial, the hypothesis was that the combination therapy of sintilimab, anlotinib, and nab-paclitaxel would achieve an ORR of at least 50% in patients with relapsed ES-SCLC. Considering that the historical ORR for topotecan, a standard second-line treatment, is around 25%, a 25% absolute improvement in ORR was deemed highly clinically meaningful for this combination therapy, thus setting the target ORR at 50%. With a target ORR of 50% for the combination therapy, enrolling 25 participants will provide approximately 80% power to demonstrate that the ORR rate of the combination therapy is greater than 25% (the historical rate for topotecan) at a significance level of one-sided 0.025. This means the lower bound of the 95% confidence interval (CI) should exclude 25%.

As appropriate, continuous variables are reported as mean (±SD) or median (with interquartile range), while categorical data are presented as counts/percentages. Results for ORR and DCR with two-sided 95% confidence intervals (CIs) were calculated using the Clopper–Pearson method, while PFS, OS, and DOR were computed via the Kaplan–Meier method. For PFS analysis, data from patients without disease progression or who remained alive were censored at their last tumor assessment. For OS analysis, data from surviving participants were censored at the date of last known contact (i.e., September 30, 2023). Statistical analysis was performed using SAS v9.4 (SAS Institute Inc., USA). Statistical tests were all two-sided at the 0.05 level of significance for superiority testing, with 95% CIs and p-values provided for inter-group comparisons. Rate comparisons employed χ2 or Fisher’s tests, and median comparisons used Wilcoxon tests. Kaplan–Meier analysis was applied to link bTMB/ctDNA with PFS; curve comparisons were made via log-rank tests. Cox regression pinpointed risk factors. These analyses were performed using R and IBM SPSS v22.0, considering *p* < 0.05 as significant.

## Supplementary information

Supplementary material

CLINICAL STUDY PROTOCOL

## Data Availability

Data are available upon request by contacting the corresponding author, Zhehai Wang (wzhai8778@sina.com). Approval from the ethics committees is required for access. Clinical data are not publicly accessible due to patient privacy concerns but can be obtained from the corresponding author for a period of three years. Individual de-identified patient data will be provided for clinical study analyses. Sequencing data have been submitted to the China National Center for Bioinformation (https://www.cncb.ac.cn/, ID: HRA008255). All other data are included in the Manuscript and Supplemental Materials.
