# Supplementary material for: Sintilimab combined with anlotinib and chemotherapy as second-line or later therapy in extensive-stage small cell lung cancer: a phase II clinical trial
Source: Signal Transduct Target Ther. 2024 Sep 16;9:241. doi: 10.1038/s41392-024-01957-3 (PMC11402985; doi:10.1038/s41392-024-01957-3)
Supplement: Supplementary file 1 — Supplementary material [file 41392_2024_1957_MOESM1_ESM.docx]

Supplementary Materials for

**Sintilimab combined with anlotinib and chemotherapy as second-line or later therapy in extensive-stage small cell lung cancer: a phase II clinical trial**

Xiao Han^a^, Jun Guo^a^, Lingyu Li^b^, Yong Huang^c^, Xue Meng^d, e^, Linlin Wang^d^, Hui Zhu^d^, Xiangjiao Meng^d^, Qian Shao^d^, Xing Li^f^, Yan Zhang^a^, Jin Wang^f^, Yanhua Chen^f^, Yingjie Zhang^d^, Yiru Chen^d^, Changbin Zhu^f^, Zhehai Wang^a*^

Correspondence to: wzhai8778@sina.com

**This PDF file includes:**

Figure S1

**Fig. S1**

**
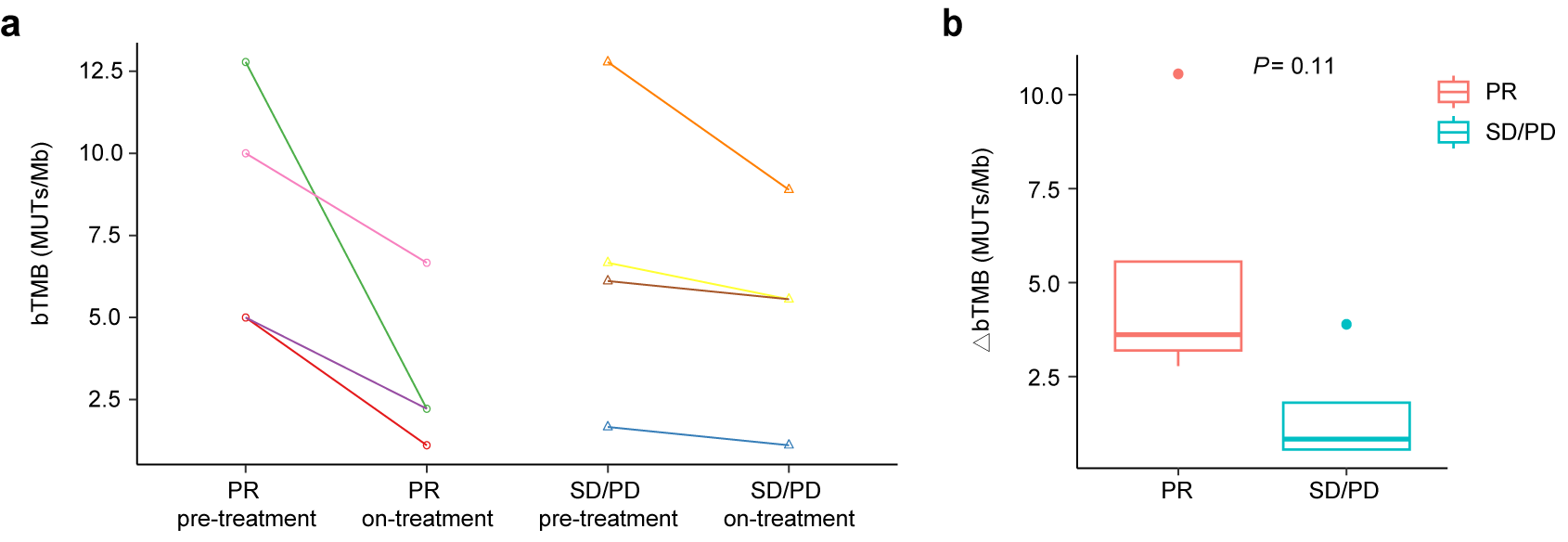
**

**Fig. S1** Dynamic changes in bTMB relative to treatment response, illustrating the relationship between changes in bTMB and treatment efficacy. **(a)** Line graph showing individual patient bTMB trajectories from pretreatment to on-treatment time points, with the distinct paths indicating more significant reductions in bTMB among those who achieved PR than for those with SD/PD. **(b)** Box plot comparing the distribution of ΔbTMB between response subgroups, highlighting a notably greater, though not statistically significant, reduction in bTMB in the PR group (p = 0.11). This observation supplements the finding that the median on-treatment bTMB value is significantly correlated with PFS and OS, underscoring the potential of bTMB as a predictive biomarker for clinical outcomes.
