# Supplementary material for: Sintilimab combined with anlotinib and chemotherapy as second-line or later therapy in extensive-stage small cell lung cancer: a phase II clinical trial
Source: Signal Transduct Target Ther. 2024 Sep 16;9:241. doi: 10.1038/s41392-024-01957-3 (PMC11402985; doi:10.1038/s41392-024-01957-3)
Supplement: Supplementary file 2 — CLINICAL STUDY PROTOCOL [file 41392_2024_1957_MOESM2_ESM.docx]

**CLINICAL STUDY PROTOCOL**

**Sintilimab Combined with Anlotinib and Chemotherapy as Second-Line or Later Therapy in Extensive-Stage Small Cell Lung Cancer: A Phase II Clinical Trial**

**Synopsis**

| **Sponsor** | Shandong Cancer Hospital and Institute |
| --- | --- |
| **Study Title** | Sintilimab Combined with Anlotinib and Chemotherapy as Second-Line or Later Therapy in Extensive-Stage Small Cell Lung Cancer: A Phase II Clinical Trial |
| **Investigational Drugs** | Recombinant fully human anti-programmed death receptor-1 (PD-1) monoclonal antibody injection (Sintilimab)  Other drugs: Albumin-Bound Paclitaxel, Anlotinib |
| **Study Phase** | Phase II, Single-Arm, Single-Center |
| **Study Objectives** | Primary Objectives  - Evaluate the objective response rate (ORR) of Sintilimab combined with Anlotinib and Albumin-Bound Paclitaxel in treating second-line and beyond extensive-stage small cell lung cancer (ED-SCLC). - Evaluate the safety and tolerability of Sintilimab combined with Anlotinib and Albumin-Bound Paclitaxel in treating second-line and beyond ED-SCLC.  Secondary Objectives  - Assess progression-free survival (PFS) according to RECIST V1.1. - Assess disease control rate (DCR) according to RECIST V1.1. - Assess duration of response (DOR) according to RECIST V1.1. - Overall survival (OS).  Exploratory Objectives:  - Explore efficacy assessment using immune Response Evaluation Criteria in Solid Tumors (iRECIST). - Explore potential predictive biomarkers for efficacy, including but not limited to PD-L1 expression levels in tumor tissue specimens and tumor mutational burden (TMB). |
| **Study Design** | This study is a single-center, prospective, single-arm Phase II clinical trial. After signing the informed consent form and meeting the inclusion criteria, subjects with extensive-stage small cell lung cancer (ED-SCLC) will receive Sintilimab combined with Anlotinib and Albumin-Bound Paclitaxel treatment for up to 6 cycles, followed by maintenance therapy with Sintilimab until disease progression (PD), intolerable toxicity, withdrawal of consent, initiation of other anti-tumor therapy, death, or other situations requiring discontinuation as per protocol, whichever occurs first, with a maximum treatment duration of 12 months. Clinical tumor imaging evaluations will be conducted using RECIST v1.1, with efficacy evaluations every 6 weeks during treatment.  In this single-arm, phase II trial, the hypothesis was that the combination therapy of Sintilimab, Anlotinib, and nab-Paclitaxel would achieve an ORR of at least 50% in patients with relapsed ED-SCLC. Considering the historical ORR for topotecan, a standard second-line treatment, is around 25%, a 25% absolute improvement in ORR was deemed highly clinically meaningful for this combination therapy, thus setting the target ORR at 50%. With a target ORR of 50% for the combination therapy, enrolling 25 participants will provide approximately 80% power to demonstrate that the ORR rate of the combination therapy is greater than 25% (the historical rate for topotecan) at a significance level of one-sided 0.025. This means the lower bound of the 95% confidence interval (CI) should exclude 25%.  Subjects receiving Sintilimab treatment who have radiographic evidence of PD according to RECIST 1.1, and are clinically stable without rapid radiographic progression, may continue current study treatment upon investigator's assessment of potential benefit, with repeat radiographic evaluation for confirmation at least 4 weeks (±7 days) later. If PD is confirmed upon reevaluation, study treatment should be discontinued; if not confirmed, continue study treatment with subsequent evaluations as per protocol until radiographic confirmation of PD. |
| **Inclusion Criteria** | Eligible subjects must meet all of the following criteria:   1. Signed written informed consent before any trial-related procedures. 2. Aged ≥18 years and ≤75 years. 3. Histologically or cytologically confirmed extensive-stage small cell lung cancer (SCLC). 4. At least one radiographically measurable lesion according to RECIST 1.1. Lesions within previously irradiated areas can be considered measurable if progression is confirmed. 5. Previously received at least one line of systemic therapy for SCLC. 6. Subjects with asymptomatic or stable brain metastases after local treatment may be included if they meet the following criteria:    1. Measurable lesions outside the central nervous system (CNS)    2. No CNS symptoms or no symptom exacerbation for at least 2 weeks    3. No need for corticosteroid treatment or stable corticosteroid dose reduced to ≤10 mg/day prednisone (or equivalent) within 7 days before enrollment 7. Palliative radiotherapy (including cranial radiotherapy for symptomatic brain metastases) is allowed but must be completed at least 1 week before the first study drug administration, with radiotherapy-related toxicity reduced to ≤ Grade 1 (CTCAE 5.0, except alopecia). 8. ECOG performance status 0-1. 9. Expected survival time >3 months. 10. Adequate organ function, as evidenced by the following laboratory parameters:     1. ANC ≥1.5×10⁹/L without granulocyte colony-stimulating factor use within the past 14 days;     2. Platelets ≥100×10⁹/L without transfusion within the past 14 days;     3. Hemoglobin >9g/dL without transfusion or erythropoietin use within the past 14 days;     4. Total bilirubin ≤1.5×ULN;     5. AST and ALT ≤2.5×ULN (≤5×ULN if liver metastases are present);     6. Serum creatinine ≤1.5×ULN and creatinine clearance ≥60 ml/min (Cockcroft-Gault formula);     7. INR or PT ≤1.5×ULN;     8. Normal thyroid function (TSH within normal range; subjects with baseline abnormal TSH but normal total T3 or FT3 and FT4 may be included);     9. Normal myocardial enzyme spectrum (isolated laboratory abnormalities deemed clinically insignificant by the investigator may be included); 11. Women of childbearing potential must have a negative urine or serum pregnancy test within 3 days prior to receiving the first study drug (Cycle 1 Day 1). If the urine test is not confirmatory, a blood test is required. Non-childbearing potential is defined as being postmenopausal for at least 1 year or having undergone surgical sterilization or hysterectomy. 12. All subjects (male or female) must use contraception with a failure rate of less than 1% during the treatment period and for 120 days (or 180 days) after the last dose of study drug. |
| **Exclusion Criteria** | Subjects meeting any of the following criteria will be excluded:   1. Previously received any anti-PD-1, anti-PD-L1, or anti-PD-L2 therapy or any other T cell co-stimulation or co-inhibition drug (e.g., CTLA-4, OX-40, CD137). 2. Diagnosed with malignancy other than SCLC within 5 years prior to the first dose (excluding treated basal cell carcinoma, squamous cell carcinoma of the skin, or in situ carcinoma). 3. Currently participating in an interventional clinical study or has received other investigational drugs or devices within 4 weeks before the first dose. 4. Active autoimmune disease requiring systemic treatment within 2 years before the first dose (excluding replacement therapy, e.g., thyroxine, insulin, or physiological corticosteroids for adrenal or pituitary insufficiency). 5. Receiving systemic corticosteroids (excluding nasal spray, inhalation, or other topical corticosteroids) or any other immunosuppressive therapy within 7 days before the first dose. Note: Physiological doses of corticosteroids (≤10 mg/day prednisone or equivalent) are allowed. 6. Clinically uncontrollable pleural effusion/ascites (not requiring drainage or no significant increase in fluid accumulation 3 days after stopping drainage). 7. Known history of allogeneic organ transplant (except corneal transplant) or allogeneic hematopoietic stem cell transplant. 8. Known allergy to Sintilimab, Anlotinib, Albumin-Bound Paclitaxel, or their excipients. 9. Not fully recovered from any toxicity and/or complications of previous interventions (≤ Grade 1, excluding fatigue or alopecia). 10. Known history of HIV infection (positive HIV 1/2 antibodies). 11. Untreated active hepatitis B (HBsAg positive and HBV-DNA detected above the normal limit of the research center's laboratory). Note: Subjects meeting the following criteria may be included:     - 1. HBV viral load <1000 copies/ml (200 IU/ml) before the first dose, receiving anti-HBV therapy during chemotherapy to prevent viral reactivation.       2. Anti-HBc(+), HBsAg(-), anti-HBs(-), and HBV viral load(-) subjects do not require preventive anti-HBV therapy but need close monitoring for viral reactivation. 12. Active HCV infection (positive HCV antibody and HCV-RNA above detection limit). 13. Vaccination with live vaccine within 30 days before the first dose. Note: Inactivated virus vaccines for seasonal influenza are allowed; live attenuated intranasal influenza vaccines are not. 14. Pregnant or breastfeeding women. 15. Any severe or uncontrolled systemic diseases, such as:     - Significant and symptomatic abnormalities on resting ECG, such as complete left bundle branch block, grade II or higher heart block, ventricular arrhythmias, or atrial fibrillation;     - Unstable angina, congestive heart failure, chronic heart failure NYHA class ≥2;     - Myocardial infarction within 6 months before enrollment;     - Uncontrolled blood pressure (SBP >140 mmHg, DBP >90 mmHg);     - Non-infectious pneumonia requiring corticosteroid treatment within the year before the first dose or currently active interstitial lung disease;     - Active tuberculosis;     - Active or uncontrolled infection requiring systemic treatment;     - Active diverticulitis, abdominal abscess, gastrointestinal obstruction;     - Liver disease such as cirrhosis, decompensated liver disease, acute or chronic active hepatitis;     - Poorly controlled diabetes (FBG >10 mmol/L);     - Urine protein ≥++, confirmed 24-hour urine protein >1.0 g;     - Mental disorder affecting compliance with treatment. 16. Any history or evidence of disease, treatment, or laboratory abnormalities that could interfere with trial results, prevent full participation, or pose unacceptable risk according to the investigator. |
| **Drug Administration** | 1. Sintilimab: 200 mg IV infusion on Day 1 of each cycle, every 3 weeks (Q3W). 2. Anlotinib: 12 mg daily on Days 1-14, every 3 weeks (Q3W), up to 6 cycles. 3. Albumin-Bound Paclitaxel: 260 mg/m² on Day 1 of each cycle, every 3 weeks (Q3W), up to 6 cycles. |
| **Evaluation Criteria** | Efficacy Evaluation: Primary and secondary efficacy endpoints will be assessed according to RECIST 1.1 by the investigator:   1. ORR: Proportion of subjects achieving complete response (CR) or partial response (PR). 2. PFS: Time from treatment start to first radiographic disease progression or death. 3. DCR: Proportion of subjects achieving CR, PR, or stable disease (SD). 4. DOR: Interval from first documentation of response to disease progression or death. 5. OS: Time from treatment start to death from any cause.  Safety Evaluation: Severity of AEs will be graded according to NCI CTCAE v5.0 during the study and follow-up. Safety endpoints include:   1. Incidence, severity, and relationship to study drugs of all AEs, treatment-emergent AEs (TEAEs), serious AEs (SAEs), and immune-related AEs (irAEs). 2. Number and proportion of subjects discontinuing treatment due to AEs. 3. Changes in vital signs, physical examination results, and laboratory parameters before, during, and after treatment.  Exploratory Endpoints:  1. Efficacy evaluation using iRECIST for iORR, iPFS, iDCR, and iDOR. 2. Explore potential predictive biomarkers for efficacy, including but not limited to PD-L1 expression and TMB levels in tumor tissue specimens. |
| **Sample Size Calculation** | In this single-arm, phase II trial, the hypothesis was that the combination therapy of Sintilimab, Anlotinib, and nab-Paclitaxel would achieve an ORR of at least 50% in patients with relapsed ED-SCLC. Considering the historical ORR for topotecan, a standard second-line treatment, is around 25%, a 25% absolute improvement in ORR was deemed highly clinically meaningful for this combination therapy, thus setting the target ORR at 50%. With a target ORR of 50% for the combination therapy, enrolling 25 participants will provide approximately 80% power to demonstrate that the ORR rate of the combination therapy is greater than 25% (the historical rate for topotecan) at a significance level of one-sided 0.025. This means the lower bound of the 95% confidence interval (CI) should exclude 25%. |
| **Study Duration** | Estimated enrollment period: 12 months  Follow-up period: 12 months |

**1. Research Background**

**1.1 Epidemiology of Small Cell Lung Cancer**

Lung cancer is the most common malignant tumor in China. According to the 2016 report by the National Cancer Center, lung cancer ranks first in incidence among male subjects and is second only to breast cancer among female subjects (1). The major types include non-small cell lung cancer (NSCLC) and small cell lung cancer (SCLC).

SCLC accounts for 10%-15% of newly diagnosed lung cancers and is a highly invasive neuroendocrine tumor that differs significantly from NSCLC in clinical and pathological characteristics. It grows rapidly, easily develops resistance, and metastasizes early. Almost all SCLC patients have a severe smoking history (2,3). Traditionally, SCLC is divided into limited-stage disease (LD-SCLC, where the tumor is confined to a single radiation field) and extensive-stage disease (ED-SCLC, where the tumor extends beyond the boundaries of a single radiation field) (4). Most patients are diagnosed at the ED-SCLC stage. Four to six cycles of platinum (cisplatin or carboplatin) combined with etoposide (EP) remain the standard first-line treatment for ED-SCLC. The ORR of platinum combined with etoposide for LD-SCLC can reach 70%-90%, and for ED-SCLC it is 60%-70%. The median OS for LD-SCLC and ED-SCLC is 14-20 months and 9-11 months, respectively. With appropriate treatment, the 2-year survival rate is 40% for LD-SCLC and less than 5% for ED-SCLC (5). Combined chest radiotherapy can improve the local control rate of LD-SCLC by 25% (6).

In China, the ALTER1202 phase II study comparing anlotinib to placebo for third-line and above treatment of SCLC showed that anlotinib extended PFS by 3.4 months (4.1 months vs. 0.7 months), reducing the risk of disease progression by 81% (7). OS also showed significant benefit, with anlotinib group at 7.3 months compared to 4.9 months in the placebo group, HR=0.53 (8). Subgroup analysis showed that in patients with brain metastasis, PFS was extended by 3 months (3.8 months vs. 0.8 months, HR 0.15), and OS was extended by 3.7 months (6.3 months vs. 2.6 months, HR 0.23) (9). The CSCO guidelines recommend anlotinib as a treatment for SCLC patients in third-line and above settings.

**1.2 Advances in Immune Checkpoint Inhibitors for Small Cell Lung Cancer**

During tumor development, tumor cells undergo many genetic and epigenetic changes compared to normal cells, theoretically presenting enough antigens to be recognized by the immune system, triggering an immune response to inhibit tumor growth. However, tumors suppress immune responses in various ways to evade immune attack (10).

Effective antitumor immunity involves T cells as core executors, recognizing antigens mediated by T cell receptors (TCR) while various co-stimulatory and co-inhibitory signals finely regulate T cell response intensity and quality (11). Among these inhibitory signals are immune checkpoints (ICIs). Physiologically, these checkpoints help maintain immune tolerance to self-antigens, preventing autoimmune diseases, and avoiding tissue damage from excessive immune activation (12). Tumor cells exploit these checkpoints to inhibit T cell activation and evade immune destruction.

In 2016, the CHECKMATE-032 study demonstrated significant survival benefits of nivolumab ± ipilimumab in recurrent SCLC treatment (13), and in 2017, NCCN recommended it as a 2A treatment for recurrent SCLC. The 2018 CHECKMATE-032 study published in JTO presented subgroup analysis results for nivolumab as third-line treatment (14), showing a median response duration of 17.9 months, median PFS of 1.4 months, 6-month PFS of 17.2%, median OS of 5.6 months, 12-month OS of 28.3%, and 18-month OS of 20.0%. Based on these results, FDA approved nivolumab in August for metastatic SCLC patients who had received platinum-based chemotherapy and at least one other treatment, marking the first FDA-approved third-line treatment for SCLC in 20 years.

Recent studies, including phase Ib (KEYNOTE-028) and phase II (KEYNOTE-158), evaluated pembrolizumab in 83 evaluable recurrent SCLC patients (15, 16). The results showed an objective response rate of 19.3%, median OS of 7.7 months (95% CI, 5.2-10.1), with higher survival and response rates in PD-L1 positive patients. Grade 3 or 4 adverse events occurred in 12% of patients, with two treatment-related deaths (pneumonitis and encephalitis). The 2019 NCCN SCLC guidelines updated to include pembrolizumab as a subsequent treatment option for SCLC patients regardless of PD-L1 expression.

The IMpower-133 study, a global phase III, double-blind, randomized, placebo-controlled trial, enrolled ED-SCLC patients with measurable lesions, comparing first-line atezolizumab combined with etoposide and carboplatin (EP) to placebo EP (201 vs. 202 patients) (17, 18). With a median follow-up of 13.9 months, primary endpoint analysis showed atezolizumab EP group extended OS by 2 months (12.3 vs. 10.3 months), reducing death risk by 30%; 12-month OS rates were 51.7% vs. 38.2%; median PFS was 5.2 months for the combination group and 4.3 months for the chemotherapy group, with 6-month PFS rates of 30.9% vs 22.4%, and 12-month PFS rates of 12.6% vs 5.4%; median duration of response was 4.2 months for the combination group and 3.9 months for the chemotherapy group, favoring the combination group. Safety analysis showed similar adverse event (AE) rates between groups, with 100% and 96.4% any-grade AE rates in the combination and chemotherapy groups respectively, and 67.2% and 63.8% grade 3-4 AE rates. The most common grade 3-4 AEs in the combination group were neutropenia, anemia, and decreased neutrophil count. On March 18, 2019, FDA approved atezolizumab (Tecentriq) combined with carboplatin and etoposide for first-line treatment of ES-SCLC patients, the first approved immunotherapy for first-line SCLC treatment.

The CASPIAN study, a global multicenter phase III randomized controlled trial, included untreated ED-SCLC patients (19), randomized into three groups: 1) Dual immunotherapy + chemotherapy: Durvalumab 1500 mg + Tremelimumab 75 mg + EP for 4 cycles, followed by maintenance Durvalumab + Tremelimumab. 2) PD-L1 monoclonal antibody + chemotherapy: Durvalumab 1500 mg + EP for 4 cycles, followed by maintenance Durvalumab 1500 mg Q4W. 3) Chemotherapy group: EP for 4 cycles. The 2019 WCLC conference presented data comparing PD-L1 monoclonal antibody + chemotherapy to standard chemotherapy, showing significantly extended median OS (13 months vs. 10.3 months, HR=0.73, P=0.0047), reducing death risk by 27%. One-year OS rates were 53.7% vs. 39.8%; 18-month OS rates were 33.9% vs. 24.7%. Median PFS was 5.1 vs. 5.4 months (HR=0.78, 95% CI 0·65–0·94); one-year PFS rates were 17.5% vs 4.7%; ORR rates were 67.9% vs 57.6%; median DOR was 5.1 vs. 5.1 months. Subgroup analysis showed OS benefits across various characteristics, with significant benefits in younger patients (<65 years), non-smokers, and those without brain metastases. Safety analysis showed grade 3-4 AE rates of 61.5% vs 62.4% for the combination and chemotherapy groups, with immune-related AE rates of 19.6% vs 2.6%. CASPIAN study results provide new options for ES-SCLC patients.

Based on the above clinical studies, PD-1/PD-L1 monoclonal antibodies have demonstrated confirmed efficacy and controllable toxicity in SCLC, with combination therapy potentially synergizing to enhance efficacy. This study aims to explore the application of PD-1 inhibitor sintilimab combined with anlotinib and nab-paclitaxel in treating extensive-stage SCLC, to evaluate its efficacy and safety, and to preliminarily compare it with current standard treatment options. This could potentially provide new treatment choices for extensive-stage SCLC, improving patient prognosis and quality of life.

**1.3 Study Drug: Sintilimab**

**1.3.1 Mechanism of Action of Sintilimab**

Sintilimab (research code: IBI308) is a recombinant fully human IgG4 monoclonal antibody targeting PD-1, developed by Innovent Biologics (Suzhou) Co., Ltd. (hereinafter referred to as “Innovent”). It specifically binds to the PD-1 molecule on the surface of T lymphocytes, blocking the PD-1/PD-L1 pathway that leads to tumor immune tolerance, thereby reactivating the antitumor activity of T lymphocytes to achieve tumor treatment. Sintilimab targets the same site as nivolumab and pembrolizumab but has a different amino acid sequence. Multiple preclinical in vitro studies have verified the effectiveness of sintilimab in blocking the PD-1 pathway. Completed preclinical pharmacodynamic, animal pharmacokinetic, and toxicological studies have shown that sintilimab has clear targets, reliable cell line sources, good drug stability, and has demonstrated good activity in completed preclinical studies. Detailed research results can be found in the investigator’s brochure.

**1.3.2 Pharmacokinetic and Pharmacodynamic Studies of Sintilimab**

In September 2016, the phase Ia dose-escalation trial (study code CIBI308A101-1a) of sintilimab was initiated. Phase Ia enrolled subjects with advanced solid tumors who failed standard treatments. The dose-escalation decision followed the classical “3+3” design, evaluating four dose levels (1 mg/kg, 3 mg/kg, 200 mg, and 10 mg/kg). After completing the 1 mg/kg dose group, subjects were randomized 1:1 to independently evaluate the 3 mg/kg and 200 mg dose groups. The DLT observation period for each dose group was 28 days after the first administration, and subjects could enter subsequent sintilimab treatments (1 mg/kg, 3 mg/kg, and 10 mg/kg every two weeks or 200 mg every three weeks) after completing the DLT observation period, until disease progression, intolerable toxicity, withdrawal of informed consent, or other reasons for discontinuation (whichever occurs first).

Pharmacokinetic studies in phase Ia showed that sintilimab injection, administered via intravenous infusion, gradually increased in blood concentration after the start of infusion, reaching Cmax after stopping infusion, and then decreased slowly. Within the 1-10 mg/kg dose range, sintilimab exposure in the body increased proportionally with dose, indicating linear kinetics. In subjects with solid tumors, the elimination half-life (Geo.Mean [CV%]) after a single dose of sintilimab was 14.4 [28.9%] days, clearance rate was 11.5 [42.5%] mL/h, steady-state distribution volume was 5.43 [34.4%] L, and apparent distribution volume was 5.77 [33.2%] L, similar to the PK characteristics of marketed anti-PD-1 antibodies (nivolumab and pembrolizumab).

In the pharmacodynamic study of phase Ia subjects with advanced solid tumors, a single dose of 1 mg/kg (N=3) of sintilimab quickly (within 24 hours) saturated (mean ≥95%) the PD-1 receptors on CD3+ T cells in the peripheral blood of subjects with solid tumors, and maintained saturation levels throughout the 28-day study period and continuous multi-dose treatment. The PD-1 occupancy results for the 3 mg/kg (N=3), 200 mg (N=3), and 10 mg/kg (N=3) dose groups were similar to those for the 1 mg/kg dose group, indicating no dose or concentration dependency within the 1-10 mg/kg dose range. Based on preliminary pharmacokinetic and pharmacodynamic results and acceptable safety events, and considering potential individual differences, the dosage for subsequent studies was set at 200 mg Q3W.

**1.3.3 Clinical Study Results of Sintilimab in Lung Cancer Subjects**

The phase 1b study CIBI308A101-1b is a multicenter clinical trial conducted in China involving subjects with advanced solid tumors. Cohort C mainly included locally advanced, recurrent, or metastatic NSCLC subjects who failed standard treatments, receiving 200 mg of sintilimab monotherapy every three weeks to evaluate the safety and tolerability of sintilimab monotherapy and preliminarily assess its antitumor activity in subjects with advanced NSCLC (20). As of September 1, 2018, 37 subjects were enrolled in this cohort, with 34 evaluable subjects. All subjects received at least one dose of sintilimab, with a median treatment cycle of 6 (1-18) cycles. Based on RECIST1.1 evaluation, the ORR was 14.7%, and the DCR was 52.9%, with 5 subjects (14.7%) achieving partial response (PR) and 13 subjects (38.2%) having stable disease (SD). Based on iRECIST evaluation, the ORR was 17.6%, and the DCR was 61.7%, with 6 subjects (17.6%) achieving PR and 15 subjects (44.1%) having SD.

The phase 1b study cohort D included subjects with advanced, recurrent, or metastatic non-squamous NSCLC who had not received systemic anticancer treatments, receiving sintilimab combined with pemetrexed and cisplatin. This cohort aimed to observe the safety and tolerability of sintilimab combined with chemotherapy in this population and preliminarily evaluate its antitumor activity (21). As of September 1, 2018, 21 subjects were enrolled and received at least one dose of the study drug, with a median treatment cycle of 10 (1-19) cycles. Nineteen subjects underwent at least one imaging evaluation. Based on existing data and RECIST 1.1 evaluation, the best overall response (BOR) results showed an ORR of 68.4%, with 13 subjects achieving PR and 3 subjects having SD, while 3 subjects experienced disease progression (PD).

The phase 1b study cohort E included subjects with advanced, recurrent, or metastatic squamous NSCLC who had not received systemic anticancer treatments, receiving sintilimab combined with gemcitabine and cisplatin. As of September 1, 2018, 20 subjects were enrolled, all of whom received at least one dose of sintilimab. Seventeen subjects completed at least one efficacy evaluation, with 11 subjects achieving PR (64.7%) and 6 subjects having SD (25.3%). The ORR was 64.7%, and the DCR was 100%, with ≥3 grade treatment-related adverse events occurring in 5.0% of subjects, demonstrating good efficacy and safety (22).

Currently, multiple phase II/III clinical studies involving sintilimab have been conducted in different tumor types. Based on the results of a multicenter, single-arm phase II clinical study (ORIENT-1) in relapsed or refractory classic Hodgkin lymphoma, sintilimab was approved by the National Medical Products Administration (NMPA) on December 24, 2018, for the treatment of relapsed or refractory classic Hodgkin lymphoma after at least two lines of systemic chemotherapy.

The exploratory studies on the efficacy of sintilimab combined with anlotinib in previously untreated, driver gene-negative stage IIIB-IV NSCLC were reported at the 2019 and 2020 WCLC conferences. The primary endpoint ORR was 72.7% (PR was 72.7%), with a DCR of 100%. The median PFS was 15 months (95% CI 8.3 months-NR), and the 12-month PFS rate was 71.4% (95% CI 47.2%-86.0%).

The results of a prospective, single-center, single-arm phase II clinical trial on the efficacy and safety of sintilimab combined with docetaxel in previously treated advanced NSCLC patients were presented at the 2020 ESMO conference. The median PFS reached 5.78 months (95% CI 4.44-NA). The ORR was 25%, including 1 CR. The DCR was 93.75%, with a median duration of response (DOR) of 6.46 months. The safety was tolerable, showing good efficacy and controllable safety of sintilimab combined with docetaxel.

**Safety Summary of Sintilimab**

The safety summary data for sintilimab come from six clinical studies conducted in China, involving tumor types such as NSCLC (N=619), classic Hodgkin lymphoma (N=96), and other malignant solid tumors (N=146). A total of 861 patients received at least one dose of sintilimab, including 520 patients treated with sintilimab monotherapy and 341 patients treated with sintilimab combined with dual chemotherapy.

Among the 520 patients treated with sintilimab monotherapy, the incidence of adverse reactions was 78.7%, with adverse reactions occurring in ≥10% of patients including abnormal thyroid function tests, fever, hypothyroidism, elevated aspartate aminotransferase (AST), elevated alanine aminotransferase (ALT), other blood routine abnormalities, anemia, and proteinuria. Grade 3 or higher adverse reactions occurred in 19.0% of patients, with adverse reactions occurring in ≥1% of patients including elevated lipase, non-infectious pneumonia, pulmonary infection, other blood routine abnormalities, and other electrolyte disturbances or acid-base imbalances.

Among the 341 patients treated with sintilimab combined with dual chemotherapy, the incidence of adverse reactions was 79.5%, with adverse reactions occurring in ≥10% of patients including elevated AST, elevated ALT, other blood routine abnormalities, anemia, leukopenia, rash, abnormal thyroid function tests, fatigue, decreased appetite, thrombocytopenia, and hypothyroidism. Grade 3 or higher adverse reactions occurred in 21.4% of patients, with adverse reactions occurring in ≥1% of patients including other blood routine abnormalities, thrombocytopenia, anemia, leukopenia, and non-infectious pneumonia.

**1.4 Study Basis and Risk/Benefit Assessment**

**1.4.1 Risk/Benefit Assessment**

Considering the mechanisms of action of sintilimab and chemotherapy, as well as clinical safety information from products with similar mechanisms, the anticipated adverse events during this study are primarily immune-related inflammations such as pneumonitis, thyroiditis, hepatitis, dermatitis/skin lesions, etc., due to immune system activation. Chemotherapy-related adverse events mainly include bone marrow suppression, nausea and vomiting, mucositis, alopecia, and neuropathy. Important adverse events from anlotinib treatment include bleeding, hypertension, myocardial ischemia, proteinuria, hand-foot syndrome, and gastrointestinal reactions. According to clinical data on PD-1 monoclonal antibody drugs and chemotherapy treatments, the overall tolerability is good. Only a small portion of subjects will discontinue treatment due to adverse events, and most adverse events are manageable and can be alleviated with appropriate treatment. Due to the variable early symptoms of immune-related adverse events, researchers must pay special attention to the early symptoms and signs of various immune-related reactions during the clinical study, make correct judgments promptly, adjust dosages, and provide effective treatments to reduce the risk to subjects. It is also important to exclude subjects with autoimmune diseases and those with bone marrow suppression to avoid exacerbation of pre-existing conditions due to immune system activation or imbalance.

Previous clinical studies of sintilimab and anlotinib have shown that immune checkpoint inhibitors and anlotinib both have clear pharmacological activity and good tolerability, and they have demonstrated efficacy in extensive-stage SCLC as monotherapies. Therefore, this study aims to provide effective treatment to ensure clinical benefits for the subjects.

This study aims to further explore the efficacy and safety of sintilimab combined with anlotinib and nab-paclitaxel in treating small cell lung cancer, potentially providing new and promising treatment options for SCLC patients, improving their prognosis, and enhancing their quality of life.

**2. Objectives of the Study**

This study is a single-center, prospective, single-arm phase II clinical trial. Subjects with small cell lung cancer will sign an informed consent form and, after screening to meet the inclusion criteria, will receive treatment with sintilimab combined with anlotinib and nab-paclitaxel until disease progression or intolerance.

**2.1 Primary Objective**

To evaluate the efficacy of sintilimab combined with anlotinib and nab-paclitaxel in second-line and above SCLC patients.

**2.2 Secondary Objective**

To evaluate the safety of sintilimab combined with anlotinib and nab-paclitaxel in second-line and above SCLC patients.

**2.3 Exploratory Objectives**

To explore the use of the immune Response Evaluation Criteria in Solid Tumors (iRECIST) for efficacy evaluation of subjects.

To explore potential biomarkers that may predict efficacy, including but not limited to PD-L1 expression levels in tumor tissue samples and tumor mutation burden (TMB).

**3．Study Design**

**3.1 Overall Study Design**

This study is a single-center, prospective, single-arm phase II study. After signing the informed consent form and screening to meet the inclusion criteria, subjects will receive treatment with sintilimab combined with anlotinib and nab-paclitaxel for up to six cycles, followed by maintenance treatment with sintilimab until disease progression (PD), intolerable toxicity, withdrawal of informed consent, initiation of other anti-tumor treatments, death, or other conditions specified in the protocol, whichever occurs first, for up to a maximum of 12 months.

The primary efficacy endpoint of this study is the objective response rate (ORR) evaluated by the investigator according to RECIST 1.1. Clinical tumor imaging evaluation will be performed using RECIST v1.1, with efficacy evaluations every 6 weeks during treatment.

Subjects receiving sintilimab treatment who show initial imaging evidence of PD according to RECIST 1.1, if clinically stable without evidence of rapid imaging progression, and the investigator believes the subject can continue to benefit from the study drug, may continue current study treatment. Imaging evaluation will be repeated after at least 4 weeks (±7 days) to confirm. If PD is confirmed upon re-evaluation, the subject should discontinue study treatment. If PD is not confirmed, study treatment will continue, with imaging evaluations at the protocol-specified time points until imaging confirms PD.

Investigators will monitor for potential adverse events (AEs) throughout the trial, grading the severity of AEs according to the guidelines of the National Cancer Institute's (NCI) Common Terminology Criteria for Adverse Events (CTCAE) version 5.0. After treatment ends, subjects will undergo a 30-day safety follow-up to monitor AEs. Serious adverse events (SAEs) will be collected within 90 days after the last dose if the subject does not receive new anti-tumor treatment. If a new anti-tumor treatment is initiated, SAEs will be collected before the new treatment starts, whichever occurs first.

This study will be conducted in accordance with Good Clinical Practice (GCP) guidelines.

**3.2 Study Endpoints**

**Primary Endpoints:**

To evaluate the objective response rate (ORR) of sintilimab combined with anlotinib and nab-paclitaxel in second-line and beyond extensive-stage small cell lung cancer (ED-SCLC).

To evaluate the safety of sintilimab combined with anlotinib and nab-paclitaxel in second-line and beyond extensive-stage small cell lung cancer (ED-SCLC).

**Secondary Endpoints:**

To evaluate progression-free survival (PFS) of subjects according to RECIST V1.1.

To evaluate the disease control rate (DCR) of subjects according to RECIST V1.1.

To evaluate the duration of response (DOR) of subjects according to RECIST V1.1.

To evaluate overall survival (OS).

To evaluate the safety and tolerability of sintilimab combined with anlotinib and nab-paclitaxel: including the incidence of adverse events (AE) and serious adverse events (SAE), and the incidence of treatment discontinuation due to AE/SAE.

**Exploratory Endpoints:**

To explore the use of the immune Response Evaluation Criteria in Solid Tumors (iRECIST) for efficacy evaluation of subjects.

To explore potential biomarkers that may predict efficacy, including but not limited to PD-L1 expression levels in tumor tissue samples and tumor mutation burden (TMB).

**4 Study Population**

**4.1 Inclusion Criteria**

Eligible subjects must meet all of the following criteria:

1. Sign written informed consent before any trial-related procedures.
2. Age ≥18 years and ≤75 years.
3. Histologically or cytologically confirmed small cell lung cancer (SCLC).
4. At least one radiographically measurable lesion according to RECIST 1.1. Lesions within a previous radiation field can be considered measurable if progression is confirmed.
5. Previously received at least one line of systemic treatment for SCLC.
6. Allow asymptomatic or symptom-stable brain metastases after local treatment, provided subjects meet the following conditions:
   1. Have measurable lesions outside the central nervous system.
   2. No central nervous system symptoms or symptoms have not worsened for at least 2 weeks.
   3. No need for corticosteroid treatment, or corticosteroid treatment stopped at least 7 days before enrollment, or corticosteroid dose stable and reduced to less than 10 mg/day prednisone (or equivalent) within 7 days before enrollment.
7. Palliative radiotherapy (including cranial radiotherapy for symptomatic brain metastases) is allowed, but radiotherapy must be completed at least 1 week before the first administration of the study drug, and radiotherapy-related toxicity must be ≤ Grade 1 (except alopecia).
8. ECOG performance status of 0-1.
9. Expected survival time >3 months.
10. Adequate organ function, meeting the following laboratory criteria:
11. Absolute neutrophil count (ANC) ≥1.5×10^9/L without using granulocyte colony-stimulating factor within the past 14 days.
12. Platelet count ≥100×10^9/L without transfusion within the past 14 days.
13. Hemoglobin >9 g/dL without transfusion or erythropoietin use within the past 14 days.
14. Total bilirubin ≤1.5 times the upper limit of normal (ULN).
15. Aspartate aminotransferase (AST) and alanine aminotransferase (ALT) ≤2.5×ULN (or ≤5×ULN for subjects with liver metastasis).
16. Serum creatinine ≤1.5×ULN and creatinine clearance (calculated by Cockcroft-Gault formula) ≥60 ml/min.
17. Normal coagulation function, defined as international normalized ratio (INR) or prothrombin time (PT) ≤1.5×ULN.
18. Normal thyroid function, defined as thyroid-stimulating hormone (TSH) within normal range. Subjects with baseline TSH outside normal range can be included if total T3 (or FT3) and FT4 are within normal range.
19. Normal cardiac enzyme profile (clinically insignificant isolated laboratory abnormalities may be allowed at the investigator's discretion).
20. Women of childbearing potential must have a negative urine or serum pregnancy test within 3 days before the first administration of the study drug (Cycle 1, Day 1). If urine pregnancy test results are inconclusive, a blood pregnancy test is required. Women of non-childbearing potential are defined as those who have been postmenopausal for at least 1 year or have undergone surgical sterilization or hysterectomy.
21. Subjects at risk of pregnancy (both male and female) must use effective contraception with a failure rate of less than 1% during the entire treatment period and for 120 days (or 180 days) after the last administration of the study drug.

**4.2 Exclusion Criteria**

Subjects meeting any of the following criteria cannot be enrolled in this study:

1. Previously received any anti-PD-1, anti-PD-L1, or anti-PD-L2 drugs, or drugs targeting other co-stimulatory or co-inhibitory T cell receptors (e.g., CTLA-4, OX-40, CD137).
2. Diagnosed with another malignant disease other than SCLC within 5 years before the first administration (excluding cured basal cell carcinoma of the skin, squamous cell carcinoma of the skin, and/or in situ carcinoma completely resected).
3. Currently participating in an interventional clinical study or received other study drugs or used study devices within 4 weeks before the first administration.
4. Active autoimmune disease requiring systemic treatment (e.g., disease-modifying drugs, corticosteroids, or immunosuppressants) within 2 years before the first administration. Replacement therapy (e.g., thyroxine, insulin, or physiologic corticosteroid replacement therapy for adrenal or pituitary insufficiency) is not considered systemic treatment.
5. Receiving systemic corticosteroid therapy (except nasal, inhaled, or other local corticosteroids) or any other form of immunosuppressive therapy within 7 days before the first administration.
   - Note: Physiologic doses of corticosteroids (≤10 mg/day of prednisone or equivalent) are allowed.
6. Clinically uncontrollable pleural or peritoneal effusion (subjects who do not require drainage or whose effusion has not significantly increased after 3 days of drainage can be enrolled).
7. Known history of allogeneic organ transplantation (except corneal transplantation) or allogeneic hematopoietic stem cell transplantation.
8. Known allergy to sintilimab, anlotinib, nab-paclitaxel, or their excipients.
9. Have not fully recovered from any toxicity and/or complications of any intervention before starting treatment (i.e., ≤ Grade 1, except fatigue or alopecia).
10. Known history of HIV infection (i.e., HIV 1/2 antibodies positive).
11. Untreated active hepatitis B (defined as HBsAg positive and HBV-DNA copies above the normal range of the laboratory center).

- Note: Subjects with the following criteria can be included:
  1. HBV viral load <1000 copies/ml (200 IU/ml) before the first administration, and subjects should receive anti-HBV treatment throughout the chemotherapy period to prevent viral reactivation.
  2. Subjects who are anti-HBc (+), HBsAg (-), anti-HBs (-), and HBV viral load (-) do not need prophylactic anti-HBV treatment but require close monitoring for viral reactivation.

1. Active HCV infection (HCV antibody positive and HCV-RNA level above detection limit).
2. Received a live vaccine within 30 days before the first administration (Cycle 1, Day 1).

- Note: Inactivated influenza vaccines for injection within 30 days before the first administration are allowed, but intranasal live attenuated influenza vaccines are not.

1. Pregnant or breastfeeding women.
2. Presence of any severe or uncontrolled systemic disease, such as:
3. Major, clinically uncontrolled abnormalities in resting ECG rhythm, conduction, or morphology, such as complete left bundle branch block, second-degree or higher heart block, ventricular arrhythmias, or atrial fibrillation.
4. Unstable angina, congestive heart failure, or chronic heart failure of NYHA grade ≥2.
5. Myocardial infarction within 6 months before enrollment.
6. Poorly controlled blood pressure (systolic blood pressure >140 mmHg, diastolic blood pressure >90 mmHg).
7. History of non-infectious pneumonitis requiring corticosteroid treatment within 1 year before enrollment or clinically active interstitial lung disease.
8. Active pulmonary tuberculosis.
9. Active or uncontrolled infection requiring systemic treatment.
10. Clinical active diverticulitis, abdominal abscess, gastrointestinal obstruction.
11. Liver diseases such as cirrhosis, decompensated liver disease, acute or chronic active hepatitis.
12. Poorly controlled diabetes (fasting blood glucose (FBG) >10 mmol/L).
13. Urine protein ≥++, and confirmed 24-hour urine protein quantification >1.0 g.
14. Mental disorders that prevent compliance with treatment.
15. Any history, disease evidence, treatment, or abnormal laboratory test values that may interfere with study results, hinder subject's full participation in the study, or indicate other conditions unsuitable for enrollment in the study.

**4.3 Restrictions During the Study**

**4.3.1 Pregnancy**

It is known that human IgG1 and IgG4 can cross the placental barrier, so medication during pregnancy is not recommended. Pregnant women cannot be enrolled in this study.

**4.3.2 Childbearing Potential**

Female subjects of childbearing potential with active sexual relationships with non-sterilized male partners, and non-sterilized male subjects with active sexual relationships with female partners of childbearing potential, must use at least one acceptable effective contraceptive method listed in Table 2 from the screening period until 180 days after the last administration of the study drug, and discuss the cessation of contraceptive measures with a responsible physician after this time. Periodic abstinence, safe period contraception, and withdrawal method are not acceptable contraceptive methods. Women of childbearing potential are defined as those who have had menarche and have not undergone sterilization (i.e., bilateral tubal ligation, bilateral salpingectomy, or hysterectomy) and have not reached menopause.

**Table 2: Effective Contraceptive Methods (at least one method must be used)**

| **Barrier Methods** | **Intrauterine Device Methods** | **Hormonal Methods** |
| --- | --- | --- |
| Male condom with spermicide | Copper T intrauterine device | Implant |
| Cervical cap with spermicide | Progesterone T intrauterine device | Hormonal contraceptive injection |
| Diaphragm with spermicide | Levonorgestrel-releasing intrauterine system (e.g., Mirena®) | Combined contraceptive pill |
|  |  | Mini-pill, contraceptive patch |

Women are considered postmenopausal if they have had 12 months of amenorrhea without an alternative medical cause, based on age requirements as follows:

If <50 years, postmenopausal is defined as 12 months or more of amenorrhea after stopping exogenous hormone therapy, with luteinizing hormone (LH) and follicle-stimulating hormone (FSH) levels in the postmenopausal range.

If ≥50 years, postmenopausal is defined as 12 months or more of amenorrhea after stopping all exogenous hormone therapy, ovarian irradiation or oophorectomy with the last menstrual period >1 year ago, chemotherapy-induced menopause with >1 year since last menstruation, or surgical sterilization (bilateral oophorectomy or hysterectomy).

**4.3.3 Breastfeeding**

It is unknown whether sintilimab is excreted in human milk. Considering that many drugs are excreted in human milk and the potential toxicity of sintilimab to infants, breastfeeding women cannot be enrolled in this study.

**4.4 Criteria for Discontinuing Treatment/Withdrawing from the Study**

**4.4.1 Discontinuing Study Treatment**

Discontinuation of study treatment does not imply withdrawal from the study. Because data on certain clinical events after treatment discontinuation may be very important for the study, these data must be collected until the subject's last scheduled visit, even if the subject has discontinued treatment. Subjects can stop treatment at any time for any reason or upon the occurrence of any adverse event, at the discretion of the investigator. Additionally, if a subject is deemed unsuitable for treatment, violates the study protocol, or for management and/or safety reasons, the investigator may discontinue the subject's treatment.

Subjects must discontinue treatment for any of the following reasons but may continue to be monitored in the study:

- The subject or the subject's legal representative requests to discontinue treatment.
- An adverse event specified in the protocol necessitates discontinuation of treatment (refer to Section 5.3.2).
- The development of another malignancy requiring active treatment.
- The occurrence of a comorbid disease that prevents further treatment.
- The investigator decides to withdraw the subject from the study.
- Positive serum pregnancy test results.
- Poor compliance by the subject.
- The investigator believes that, based on the subject's disease condition or personal circumstances, continuing study medication would place the subject at unnecessary risk.

Subjects who discontinue treatment but remain in the study for visits should complete all visits and procedures listed in the study flowchart (Table 1).

**4.4.2 Withdrawing from the Study**

Subjects must withdraw from the study if they or their legal representative withdraws consent to participate in the study. If subjects withdraw from the study, they will no longer receive treatment or attend planned visits. With the subject's consent, they can be followed up for survival after withdrawal from the study. If a subject is lost to follow-up, they must be withdrawn from the study.

**4.4.3 Clinical Criteria for Prematurely Terminating the Study**

The study will be prematurely terminated if any of the following criteria are met:

- Poor quality and quantity of data recording.
- Poor compliance with the study protocol and regulatory requirements.
- The incidence or severity of adverse drug reactions in this or other studies suggests potential harm to subject health.
- Planned changes or discontinuation of study drug development.

If it is decided to no longer provide the study drug, adequate notice will be given to make appropriate adjustments to the subject's treatment.

**5 Study Treatment**

**5.1 Study Drugs and Administration Plan**

The study drugs in this research are sintilimab, anlotinib, and nab-paclitaxel. Administration of the study drugs will begin on the first day of the first cycle (Cycle 1, Day 1) after completing the pre-dosing evaluation. Study drugs will be administered on Day 1 of each cycle, and for management reasons, dosing can occur within 3 days before or after the planned Day 1 of each cycle at the investigator's discretion.

**Table 3: Study Treatment Drugs and Administration Plan**

| **Drug** | **Dose** | **Frequency** | **Route** | **Treatment Cycle** |
| --- | --- | --- | --- | --- |
| Sintilimab | 200 mg | Q3W | IV infusion | Every 21 days, administered on Day 1 |
| Anlotinib | 12 mg | Q3W | Oral | Every 21 days, continuous use on Days 1-14 |
| Nab-paclitaxel | 260 mg/m² | Q3W | IV infusion | Every 21 days, administered on Day 1 |

Sintilimab should be infused before administering chemotherapy drugs.

**5.2 Use of Study Drugs**

**5.2.1 Sintilimab**

Sintilimab used in this study is provided by Innovent Biologics (Suzhou) Co., Ltd., with a specification of 100 mg/vial. The main active ingredient is a recombinant fully human anti-PD-1 monoclonal antibody, with a concentration of 10 mg/mL. The solution is clear, colorless, free of foreign matter, flocculent, and precipitation. Excipients include 140 mmol/L mannitol, 25 mmol/L histidine, 20 mmol/L sodium citrate dihydrate, 50 mmol/L sodium chloride, 0.02 mmol/L disodium edetate (EDTA), 0.2 mg/mL polysorbate 80, and pH 6.0.

The smallest packaging unit is a box containing one vial of sintilimab injection. The box is labeled with the drug name, dosage form, specification, drug code, batch number, expiration date, storage conditions, and sponsor information. The vial label contains the same information but excludes dosage form, precautions, and usage instructions. All labels indicate "For Clinical Research Use Only." The product should be stored protected from light at 2-8°C with a shelf life of 24 months. If the injection appears cloudy or precipitated, it should be sealed immediately and the manufacturer notified.

Sintilimab should be infused intravenously over 30-60 minutes. It should not be administered by IV push or bolus. The dilution and infusion instructions are as follows:

- **Solution Preparation and Infusion**:
  - Do not shake the vial.
  - Allow the vial to reach room temperature (≤25°C) before use.
  - The vial can be kept at room temperature (≤25°C) for up to 24 hours after being removed from the refrigerator before dilution.
  - Visually inspect the injection for particulate matter and discoloration before administration. If visible particles are observed, discard the vial.
  - Draw 2 vials of the injection (200 mg) and transfer them into an IV infusion bag containing 0.9% sodium chloride solution to prepare a final concentration range of 1.5-5.0 mg/mL. Gently invert the bag to mix the solution.

From a microbiological standpoint, the solution should be used immediately after dilution and should not be frozen. Stability studies have shown that the diluted solution can be stored at 2-8°C protected from light for up to 24 hours, including up to 6 hours at room temperature (20-25°C) under indoor lighting. Before use, the vial and/or IV bag should be allowed to reach room temperature.

The infusion set must be equipped with a sterile, pyrogen-free, low-protein-binding in-line filter (0.2 μm). Infusion time should be within 30-60 minutes. Do not administer other drugs through the same infusion line.

This product is for single use only. Any unused drug should be discarded.

**5.2.2 Anlotinib Hydrochloride**

Anlotinib hydrochloride should be administered at 12 mg once daily, orally, before breakfast, for 2 consecutive weeks followed by a 1-week break, constituting a 21-day cycle. Treatment continues until disease progression/death/intolerable toxicity. If a dose is missed and the time to the next dose is less than 12 hours, the missed dose should not be made up.

**5.2.3 Nab-paclitaxel**

Nab-paclitaxel should be administered at a dose of 260 mg/m² by IV infusion over 30 minutes every 3 weeks. No premedication is required before administering the drug. The reconstituted solution should be milky white and homogeneous without visible particles. If particles are observed, gently invert the vial to ensure complete dispersion before administration. If precipitate is present, discard the solution. Any drug used via IV infusion should be inspected visually for particulate matter and discoloration before administration.

**5.3 Dose Adjustment**

**5.3.1 General Principles**

If adverse events occur during the study, the investigator should first determine the likely causative drug and adjust the dosage according to the severity of adverse events in the previous dosing cycle. Before each Cycle 1 Day 1 administration of the study drug, the subject's hematological, liver, and kidney function must meet the dosing criteria, and all other toxic reactions must have resolved to CTCAE Grade 0-1 or baseline levels (excluding alopecia, fatigue, specific protocol exceptions, or clinically insignificant conditions determined by the investigator). If the subject does not meet dosing criteria within the planned dosing interval due to adverse events, the next dose may be delayed. If toxicity related to a specific study drug, treatment-related adverse event, or other reasons require suspension or permanent discontinuation of these study drugs, other study drugs (e.g., sintilimab) can be administered alone if the dosing criteria are met.

All dose adjustment and treatment decisions should be recorded, including the reasons and handling.

**5.3.2 Adjustment of Sintilimab Dosage**

Throughout the study, dose adjustments for Sintilimab are not allowed. The principles for suspending and permanently discontinuing Sintilimab are outlined in Table 4.

**Table 4: Sintilimab Dose Adjustment Plan**

| **Sintilimab-Related Adverse Events** | **Severity** | **Dose Adjustment** |
| --- | --- | --- |
| **Pneumonia** | Grade 2 | Suspend treatmenta |
|  | Grade 3 or 4, or recurrent Grade 2 | Permanently discontinue |
| **Diarrhea/Colitis** | Grade 2 or 3 | Suspend treatmenta |
|  | Grade 4 | Permanently discontinue |
| **Hepatitis** | For subjects with baseline normal ALT, AST, or TBIL: Grade 2 AST, ALT (3-5x ULN) or TBIL (1.5-3x ULN) increase; for subjects with baseline AST, ALT, or TBIL > ULN: AST, ALT, or TBIL increase ≥50% (meeting Grade 2 requirements) and duration <7 days | Suspend treatmenta |
|  | For subjects with baseline normal ALT, AST, or TBIL: Grade 3 or 4 AST, ALT (>5x ULN) or TBIL increase (>3x ULN); for subjects with baseline AST, ALT, or TBIL > ULN: AST, ALT, or TBIL increase ≥50% (meeting Grade 3 or 4 requirements) and duration ≥7 days | Permanently discontinue |
| **Nephritis** | Grade 2 or 3 serum creatinine increase | Suspend treatmenta |
|  | Grade 4 serum creatinine increase | Permanently discontinue |
| **Endocrine Diseases** | Symptomatic Grade 2 or 3 hypothyroidism, Grade 2 or 3 hyperthyroidism, Grade 2 or 3 hypophysitis, Grade 2 adrenal insufficiency, Grade 3 hyperglycemia or Type 1 diabetes | Suspend treatmentb |
|  | Grade 4 hypothyroidism, Grade 4 hyperthyroidism, Grade 4 hypophysitis, Grade 3 or 4 adrenal insufficiency, Grade 4 hyperglycemia or Type 1 diabetes | Permanently discontinue |
| **Skin Adverse Reactions** | Grade 3 | Suspend treatmenta |
|  | Grade 4, Stevens-Johnson Syndrome (SJS) or Toxic Epidermal Necrolysis (TEN) | Permanently discontinue |
| **Thrombocytopenia** | Grade 3 | Suspend treatmenta |
|  | Grade 4 | Permanently discontinue |
| **Other Immune-Related Adverse Reactions** | Grade 3 or 4 elevated amylase or lipase, Grade 2 or 3 pancreatitis, Grade 2 myocarditisc, Grade 2 or 3 first occurrence of other immune-related adverse reactions | Suspend treatmenta |
|  | Grade 4 pancreatitis or any recurrent pancreatitis, Grade 3 or 4 myocarditis, Grade 3 or 4 encephalitis, Grade 4 first occurrence of other immune-related adverse reactionsd | Permanently discontinue |
| **Recurrent or Persistent Adverse Reactions** | Recurrent Grade 3 or 4 (excluding endocrine diseases), Grade 2 or 3 adverse reactions within 12 weeks after the last dose, adverse reactions not improved to 0-1 Grade (excluding endocrine diseases), corticosteroids not reduced to ≤10mg/day prednisone equivalent within 12 weeks after the last dose | Permanently discontinue |

a: Resume treatment after symptoms improve to 0-1 Grade or baseline level. *The safety of restarting Sintilimab treatment after myocarditis improves to 0-1 Grade with treatment is uncertain. b: In cases of hypophysitis, adrenal insufficiency, hypothyroidism/hyperthyroidism, and Type 1 diabetes, treatment can be resumed if adequately controlled with only physiological hormone replacement therapy. c: The safety of restarting Sintilimab treatment after myocarditis improves to 0-1 Grade with treatment is uncertain. d: For Grade 4 laboratory abnormalities, the decision to discontinue should be based on accompanying clinical symptoms/signs and the investigator's clinical judgment.

If treatment-related adverse reactions do not recover to Grade 0-1 or baseline level within 6 weeks after the last dose of Sintilimab, Sintilimab should be permanently discontinued. The maximum suspension time for Sintilimab due to corticosteroid use for treatment-related immune adverse reactions should not exceed 12 weeks. For subjects who exceed this maximum time interval, consultation with the sponsor is required before permanently discontinuing Sintilimab.

**5.3.3 Management of Sintilimab-Related Infusion Reactions**

Sintilimab may cause severe or life-threatening infusion reactions, including severe hypersensitivity or allergic reactions. Signs and symptoms typically appear during or shortly after infusion and usually resolve completely within 24 hours. The management guidelines for Sintilimab-related infusion reactions are outlined in Table 5.

**Table 5: Management Guidelines for Sintilimab Infusion Reactions**

| **NCI CTCAE Grade** | **Treatment** | **Pre-treatment for Subsequent Infusions** |
| --- | --- | --- |
| **Grade 1** | Mild reaction; infusion not interrupted; no intervention required | Monitor vital signs based on medical indication until the investigator deems the subject stable |
| **Grade 2** | Requires treatment or infusion interruption but quickly responds to symptomatic treatment (e.g., antihistamines, NSAIDs, anesthetics, IV fluids); requires ≤24 hours of preventive medication | Stop infusion and monitor symptoms. Other appropriate treatments may include but are not limited to: IV fluids, antihistamines, NSAIDs, acetaminophen, anesthetics. Monitor vital signs based on medical indication until the investigator deems the subject stable. If symptoms resolve within 1 hour after stopping infusion, resume infusion at 50% of the original rate (e.g., from 100 mL/h to 50 mL/h). Otherwise, pause treatment until symptoms resolve, and pre-treat the subject before the next scheduled dose. Subjects who experience Grade 2 toxicity despite adequate pre-treatment should permanently discontinue the study drug |
| **Grade 3 or 4** | Grade 3: prolonged duration (i.e., symptoms do not quickly respond to symptomatic medication and/or brief infusion interruption); symptoms recur after initial improvement; hospitalization required due to other clinical sequelae (e.g., renal damage, lung infiltration). Grade 4: life-threatening; requires vasopressor or ventilatory support | Stop infusion. Other appropriate treatments may include but are not limited to: epinephrine**, IV fluids, antihistamines, NSAIDs, acetaminophen, anesthetics, oxygen, vasopressors, corticosteroids. Monitor vital signs based on medical indication until the investigator deems the subject stable. Hospitalization may be required. **For anaphylaxis, use epinephrine immediately |
| **Note** | Appropriate emergency equipment should be available in the ward, and a physician should be on call during administration | For further information, refer to the Common Terminology Criteria for Adverse Events (CTCAE) Version 5.0 ([http://ctep.cancer.gov](http://ctep.cancer.gov/)) |

**5.3.4 Other Permitted Dose Adjustments for Sintilimab**

Apart from treatment-related adverse events, Sintilimab treatment may be interrupted for unrelated medical/surgical events or management reasons. Subjects should resume study treatment within 3 weeks after the planned interruption unless otherwise discussed with the sponsor. Reasons for treatment interruption should be documented in the subject's study records.

**5.3.5 Dose Adjustments for Other Medications**

**Albumin-bound Paclitaxel Dose Adjustments**: If severe neutropenia (ANC < 500/mm³ lasting 1 week or longer) or severe sensory neurotoxicity occurs, subsequent doses should be reduced to 220 mg/m². If severe neutropenia or sensory neurotoxicity occurs again, reduce subsequent doses to 180 mg/m². Treatment should be paused for patients experiencing Grade 3 sensory neurotoxicity until neurotoxicity recovers to ≤ Grade 2, after which treatment may continue at a reduced dose.

**Anlotinib Dose Adjustments**: Monitor for adverse reactions during Anlotinib use, adjusting the dose to ensure patient tolerance. Adverse reactions may be managed through symptomatic treatment, dose interruption, and/or dose adjustments. Based on the severity of adverse reactions, dose adjustments under physician guidance are recommended:

1. First dose adjustment: 10 mg once daily for 2 weeks, then 1 week off.
2. Second dose adjustment: 8 mg once daily for 2 weeks, then 1 week off. If the 8 mg dose remains intolerable, discontinue use permanently.

**5.4 Principles for Managing Toxicity of Immune Checkpoint Inhibitors**

Adverse events (AEs) associated with exposure to Sintilimab may be of immunological origin. This is due to the mechanism of action of Sintilimab, which involves blocking the interaction between PD-1 and PD-L1, thereby restoring T-cell activity. This can lead to heightened autoimmune function, resulting in immune-related adverse events (irAEs). These irAEs may occur shortly after the first dose or several months after the last dose of Sintilimab and can simultaneously affect multiple body systems, such as immune-related pneumonia, diarrhea/enterocolitis, renal dysfunction, rash, hepatitis, endocrine diseases, and peripheral or central neuritis. Therefore, early detection and initiation of treatment are crucial to reducing complications.

Based on existing clinical trial data, most irAEs are reversible and can be managed by interrupting Sintilimab treatment, administering corticosteroids, and/or other supportive therapies. If subjects in this study experience the aforementioned AEs, their symptoms and signs should be monitored, and relevant examinations such as bronchoscopy, endoscopy, or skin biopsy should be conducted to identify the cause. If no alternative cause (e.g., disease progression, concomitant medication, and infection) is found and corticosteroids and/or other immunosuppressive treatments are required (excluding endocrine events like hyperthyroidism/hypothyroidism, hypophysitis, type 1 diabetes, and adrenal insufficiency which might not require immunosuppressive therapy but are still considered related to autoimmune hyperfunction induced by Sintilimab), the AE should be considered related to the autoimmune hyperfunction induced by Sintilimab and diagnosed as an irAE.

Depending on the severity of the irAE, Sintilimab treatment should be paused or permanently discontinued, and corticosteroids should be administered.

**5.5 Concomitant Medications and Supportive Care**

**5.5.1 Permitted Concomitant Medications/Supportive Care**

- Medications deemed appropriate by the investigator according to the protocol (e.g., those for treating symptoms related to the disease and supportive care for various treatment-related adverse events).
- Long-term medications for underlying conditions such as hypertension and diabetes may be continued.
- Supportive care for alleviating tumor-related symptoms is permitted, such as bisphosphonate treatment for bone metastases.
- Local corticosteroid treatments such as topical, ophthalmic, nasal spray, and inhaled steroids are allowed.

**5.5.2 Prohibited Concomitant Medications/Supportive Care**

Subjects are prohibited from receiving the following treatments during the study:

- Biologic treatments with antitumor effects (except cytokines for treating adverse events caused by chemotherapy), as well as traditional Chinese medicines with antitumor effects.
- Immunomodulatory drugs, including but not limited to nonspecific immunomodulators (e.g., thymosin, interferons, interleukins, immunoglobulins, gamma globulins) and traditional Chinese medicines with immunomodulatory effects.
- Chemotherapy not specified in the protocol.
- Live vaccines within 30 days before the first administration of sintilimab and during the study. Live vaccines include, but are not limited to, measles, mumps, rubella, varicella, yellow fever, rabies, BCG, and oral typhoid vaccine. Injectable inactivated virus vaccines for seasonal influenza are allowed, but intranasal attenuated live influenza vaccines are not.
- Corticosteroids. Inhaled corticosteroids for treating asthma or chronic obstructive pulmonary disease (COPD) as part of a fixed regimen are allowed. Corticosteroids for managing immune-related adverse events are permitted. Physiological doses of corticosteroids can be approved after consulting the sponsor.Note: Prophylactic corticosteroids to prevent allergic reactions (e.g., premedication before IV contrast or chemotherapy) are allowed.

Subjects requiring any of the above treatments should be excluded from the trial. Subjects may receive other medically necessary treatments as deemed by the investigator. It is important for the investigator to review all medications (prescription and over-the-counter) taken by the subject before starting the study and at each study visit.

- At each visit, inquire about any new medications the subject is taking.
- To minimize the risk of adverse drug interactions, limit the number of concomitant medications to those truly necessary.
- During the administration period, avoid hepatotoxic medications (i.e., those with hepatotoxic warnings in their product labeling). Investigators are encouraged to review potential hepatotoxic drugs by searching [www.livertox.nih.gov](http://www.livertox.nih.gov/).

**5.6 Study Drug Management**

**5.6.1 Storage and Management of Study Drugs**

The study drugs for this trial are provided by the sponsor. All investigational drugs provided by the sponsor can only be used for this research and must not be used for purposes outside the protocol. Investigators must commit not to provide the study drugs to anyone not related to this trial.

Sintilimab should be refrigerated at 2-8°C, protected from light and moisture, and not frozen. All study sintilimab will be transported via cold chain to each research center. Each center should designate personnel responsible for storing and dispensing the study drug.

Study sintilimab should be stored in a refrigerator that can only be accessed by authorized personnel. Upon receipt, investigators should confirm that the transportation temperature was within the specified range, sign for the delivery, and store it at the required temperature. If there is any temperature deviation during transportation or storage, the drug should be isolated and transferred to the appropriate temperature environment without using it on subjects, and the sponsor should be notified promptly for further instructions.

**5.6.2 Drug Disposal**

Used containers of study drugs in this study can be destroyed locally according to applicable guidelines and procedures established by the research center or local institution. All unused study drugs should be collected and destroyed when the study is completed/terminated or after their expiration date.

**5.6.3 Study Drug Records**

Designated personnel at the research center should maintain timely records of the receipt, distribution, use, inventory, destruction, return, and loss of study drugs according to relevant regulations and guidelines.

**6 Study Procedures**

**6.1 Subject Screening Procedures**

**6.1.1 Subject Screening**

The investigator will enroll subjects following these steps:

1. Obtain written informed consent from the subject before any study-related procedures.
2. The investigator or trained designated personnel will review inclusion/exclusion criteria and formally determine the subject's eligibility.

**6.1.2 Handling of Ineligible Enrolled Subjects**

Strict adherence to inclusion/exclusion criteria is necessary. If a subject not meeting the criteria is enrolled, the investigator should discuss whether the subject should continue in the study. If the investigator deems it medically appropriate for the subject to continue, the subject may remain in the study and receive the study drug.

**6.2 Study Plan and Schedule**

**6.2.1 Screening Period**

The screening period (Day -28 to -1) must include the following procedures to ensure subject eligibility:

- Obtain written informed consent.
- Verify inclusion/exclusion criteria.
- Record demographic data, medical history, and previous lung cancer treatment history.
- Record previous and concomitant medications.
- Record vital signs, height, and weight.
- Perform a physical examination.
- Assess ECOG performance status.
- Conduct a 12-lead ECG.
- Perform complete blood count (CBC)/blood chemistry/urinalysis (within 7 days before the first dose).
- Assess coagulation function (within 7 days before the first dose).
- Conduct pregnancy test (within 3 days before the first dose).
- Assess thyroid function (within 28 days before the first dose).
- Assess cardiac enzyme profile (within 28 days before the first dose).
- Conduct virological antibody testing: HIV antibody, hepatitis B serology (HBsAg, HBsAb, HBcAb, HBeAg, HBeAb), and HCV antibody (within 28 days before the first dose).
- Assess HBV-DNA and HCV-RNA (if applicable) (within 28 days before the first dose).
- Evaluate adverse events.
- Record concomitant medications.
- Conduct tumor imaging assessment (within 28 days before the first dose).
- Collect archived or fresh tumor tissue samples.

**6.2.2 Treatment Period Visits**

- Record vital signs and weight.
- Assess ECOG performance status.
- Conduct a 12-lead ECG.
- Perform CBC/blood chemistry/urinalysis.
- Assess thyroid function.
- Assess cardiac enzyme profile.
- Assess HBV-DNA and/or HCV-RNA (if applicable).
- Evaluate adverse events.
- Record concomitant medications.
- Conduct tumor imaging assessment.
- Administer study drugs.
- Collect biomarker whole blood samples.

Refer to Table 1 for the treatment period visit flowchart. For detailed descriptions of tumor imaging assessments and safety evaluations, see Sections 7.1-7.2.

**6.2.3 Safety Follow-up Visits**

A safety follow-up will be conducted 30 (±7) days after the last administration of the study drug or before the initiation of new anti-tumor treatment (whichever occurs first), including the following:

- Record vital signs.
- Measure weight.
- Perform physical examination.
- Assess ECOG performance status.
- Conduct a 12-lead ECG.
- Perform CBC/blood chemistry/urinalysis.
- Assess coagulation function.
- Assess thyroid function.
- Assess cardiac enzyme profile.
- Assess HBV-DNA and/or HCV-RNA (if applicable).
- Evaluate adverse events.
- Record subsequent anti-tumor treatments (if applicable).

All adverse events occurring before the safety follow-up visit should be recorded until they resolve to Grade 0-1 or baseline levels, or until the investigator reasonably determines that follow-up is no longer needed (whichever occurs first).

**6.2.4 Survival Follow-up Visits**

After the safety follow-up, subjects enter the survival follow-up phase. Contact subjects every 90 (±7) days (telephone visits are acceptable) to obtain information related to survival and any subsequent systemic anti-tumor treatments. For subjects who discontinued study drug treatment for reasons other than disease progression, attempt to obtain information related to disease progression. Long-term follow-up will continue until the subject dies or the study ends.

Note: If a subject does not have a 30-day safety follow-up visit, survival follow-up should begin from the end of treatment.

**6.2.5 Subsequent Anti-tumor Treatments**

The investigator or qualified designated personnel should review all new anti-tumor treatments initiated after the last administration of the study drug. If a subject starts new cancer treatment within 30 days after the last administration, a safety follow-up visit must be conducted before the first dose of the new treatment. After starting new cancer treatment, the subject will enter survival follow-up.

**6.3 Additional Procedures**

**6.3.1 Discontinuation of Treatment/Withdrawal from Study**

Subjects who discontinue treatment/withdraw from the study before completing the treatment specified in the protocol should be encouraged to continue follow-up and complete all remaining study visits.

Upon discontinuation of treatment/withdrawal from the study, all applicable procedures at the end of treatment should be completed. Follow-up of any adverse events present at the time of discontinuation/withdrawal should be conducted according to the safety requirements summarized in Section 8.4. If a subject discontinues treatment/withdraws from the study for reasons other than disease progression, tumor imaging assessment should be performed at the end of treatment.

**6.3.2 Lost to Follow-up**

If a subject fails to return for required study visits and/or the study center cannot contact the subject, the following procedures should be performed:

- The study center should attempt to contact the subject and reschedule the missed visit. If the subject is contacted, they should be informed of the importance of adhering to the visit schedule.
- At each missed visit, the investigator or designated personnel should make every effort to re-establish contact with the subject (e.g., phone calls and/or sending registered letters to the last known address). These contact attempts should be documented in the subject's medical records.
- Note: A subject is not considered lost to follow-up until the last scheduled visit has been reached. The amount of missing data for the subject should be managed according to predefined data handling and analysis guidelines.

**7 Study Assessments**

**7.1 Efficacy Assessments**

**7.1.1 Tumor Imaging and Disease Assessment**

The first tumor imaging assessment during screening must be performed within 28 days before the start of treatment. Clinical routine imaging assessments, if they meet diagnostic quality standards and are performed within 28 days before treatment, can be used for screening tumor assessments.

The method used for tumor burden assessment at baseline must be consistent with the method used at each follow-up assessment (CT/MRI). Imaging of suspected involved areas (e.g., the brain) may be performed based on clinical symptoms and signs. PET/CT scans can be used for baseline assessments, and any abnormalities should be followed up with appropriate CT/MRI monitoring for subsequent evaluations.

**7.1.2 Baseline Tumor Imaging Assessment**

The first tumor imaging assessment during screening must be performed within 28 days before treatment. Before treatment, the investigator at the research center should confirm that the subject has measurable lesions according to RECIST 1.1 standards.

Clinical routine imaging assessments, if they meet diagnostic quality standards and are performed within 28 days before treatment, can be used for screening tumor assessments.

The method used for tumor burden assessment at baseline must be consistent with the method used at each follow-up assessment (CT/MRI). Imaging of suspected involved areas (e.g., the brain) may be performed based on clinical symptoms and signs.

**7.1.3 Tumor Imaging Assessment During the Study**

Throughout the study, imaging assessments should be performed according to the protocol-specified schedule. From the start of the first administration, tumor imaging assessments should be performed every 6 weeks (±7 days). For subjects with initial imaging evidence of disease progression (PD) based on RECIST 1.1, if the clinical condition is stable without evidence of rapid imaging progression and the investigator believes the subject can continue to benefit from the study drug, current study treatment can continue. Imaging assessment should be repeated at least 4 weeks (±7 days) later to confirm. If PD is confirmed upon reassessment, the subject should discontinue study treatment. If progression is not confirmed, study treatment should continue, and imaging assessments should follow the protocol-specified schedule until imaging confirms PD. If initial imaging shows PD and clinical disease is unstable, confirmation is not required 4-6 weeks later, and study treatment should be stopped.

According to iRECIST (2017), when subsequent tumor assessments after initial imaging show PD, lesions should be compared with the baseline at initial PD. If any of the following criteria are met, it is confirmed imaging progression:

- The sum of the longest diameter of target lesions increases by >5 mm.
- Further progression of non-target lesions.
- The sum of the longest diameter of new lesions from previous PD increases by >5 mm (target lesions) or shows further progression (non-target lesions).
- More new lesions appear.

If a subject discontinues study treatment for reasons other than objective disease progression, tumor imaging should be performed at the time of treatment discontinuation and should continue according to the protocol-specified imaging schedule until one of the following events occurs: initiation of new anti-tumor treatment, objective disease progression, loss to follow-up, or death, whichever occurs first.

Subjects known or suspected to have brain metastases at screening should undergo baseline brain CT/MRI before starting study treatment. During the study, these subjects should have brain CT/MRI assessments, with brain metastases evaluated as non-target lesions.

If the investigator is unable to determine disease progression, especially when assessing non-target lesions and new lesions, the subject may continue treatment. Imaging should be reassessed when clinical symptoms appear or at the next scheduled evaluation. If progression is confirmed, the date of initial detection should be recorded.

Tumor imaging assessments in this study will be conducted by the investigator based on RECIST 1.1 (see Appendix 3).

**7.1.4 Tumor Imaging Assessment at End of Treatment and Follow-up Period**

Subjects who complete treatment or discontinue treatment for reasons other than disease progression should have a tumor imaging assessment at the end of treatment/discontinuation. Imaging assessments should continue according to the protocol-specified schedule until one of the following occurs: initiation of new anti-tumor treatment, objective disease progression, death, or study end, whichever occurs first.

**7.2 Safety Assessments**

Investigators or qualified designated personnel will evaluate adverse events (AEs) for each subject during the study and follow-up periods according to the trial flowchart. AEs will be graded and recorded according to NCI CTCAE version 5.0. The characteristics of AEs will be determined based on severity, causality, toxicity grade, and actions taken concerning trial treatment.

All unknown-cause AEs occurring during study treatment should be evaluated to determine if they are immune-related AEs.

For detailed descriptions of AE evaluation and recording, refer to Section 8.

**7.2.1 Physical Examination**

**7.2.1.1 Comprehensive Physical Examination**

The investigator or qualified designated personnel will perform a comprehensive physical examination during screening, recording clinically significant abnormal results. Comprehensive physical examinations should also be conducted according to the trial flowchart schedule. Clinically significant new abnormalities found after the first study drug administration will be recorded as AEs.

**7.2.1.2 Targeted Physical Examination**

For cycles where a comprehensive physical examination is not required, the investigator or qualified designated personnel will perform targeted physical examinations based on clinical indications before each treatment cycle Day 1 administration. Clinically significant new abnormalities should be recorded as AEs.

**7.2.1.3 Height, Weight, and Vital Signs**

The investigator or qualified designated personnel will record vital signs at screening, before each treatment administration, and at the end of treatment according to the trial flowchart. Weight should be measured before each scheduled treatment during the study. If the subject's weight fluctuates by less than 10% from the baseline (first study drug administration day), the baseline weight will be used to calculate the chemotherapy drug dose. Otherwise, the actual weight on the scheduled treatment day will be used to calculate the dose.

Height should only be measured at baseline. Vital signs include body temperature, pulse, respiratory rate, and blood pressure.

**7.2.1.4 12-lead ECG**

A standard 12-lead ECG will be recorded during screening according to local standard procedures. Clinically significant abnormal results should be recorded. Additional ECG examinations may be performed at other time points based on clinical need.

**7.2.1.5 ECOG Performance Status**

The investigator or qualified designated personnel will assess ECOG performance status at screening, before each treatment cycle Day 1 administration, at the end of treatment, and during safety follow-up according to the trial flowchart.

**7.2.2 Laboratory Assessments**

Laboratory assessments include various tests as detailed below. Procedures for collecting fluid samples are outlined in the study procedure manual. Laboratory assessment timing is referenced in the trial flowchart.

**7.2.2.1 Laboratory Safety Evaluations (CBC, Coagulation Panel, Urinalysis, Blood Chemistry)**

Laboratory tests for CBC, coagulation panel, urinalysis, and blood chemistry are listed in Table 9.

**Table 9: Laboratory Assessments**

| **CBC and Coagulation Panel** | **Blood Chemistry** | **Urinalysis** | **Other** |
| --- | --- | --- | --- |
| Hemoglobin | Albumin | pH | Pregnancy test (blood or urine)a |
| Platelet count | Alkaline phosphatase | Glucose | Total triiodothyronine (T3) or FT3, FT4, and TSHb |
| WBC (total and differential)c | Alanine aminotransferase (ALT) | Protein d | Anti-HCV antibody |
| RBC | Aspartate aminotransferase (AST) | Specific gravity | HCV-RNA |
| Hematocrit | Calcium | Leukocytes | HBsAg |
| INR | Chloride | RBCs | HBV-DNA |
| PT | Creatinine |  | HBcAb |
|  | Blood glucose |  | HBeAg |
|  | Phosphorus |  | HBsAb |
|  | Magnesium |  | HBeAb |
|  | Potassium |  | HIV antibody |
|  | Sodium |  | Cardiac enzyme profile |
|  | Total bilirubin |  |  |
|  | Direct bilirubin |  |  |
|  | Total protein |  |  |
|  | Blood urea nitrogen (BUN) |  |  |
|  | Amylase |  |  |
|  | Lactate dehydrogenase (LDH) |  |  |
|  | Gamma-glutamyl transferase (γ-GT) |  |  |
|  | Creatine kinase |  |  |

**Abbreviations:** FT4 = free thyroxine; HBc = hepatitis B core antigen; HBeAg = hepatitis B e antigen; HBeAb = hepatitis B e antibody; HBV = hepatitis B virus; HBsAb = hepatitis B surface antibody; HBsAg = hepatitis B surface antigen; HCV = hepatitis C virus; INR = international normalized ratio; PT = prothrombin time; RBC = red blood cells; T3 = total triiodothyronine; TSH = thyroid-stimulating hormone; WBC = white blood cells.

- **a:** Applicable only for females of childbearing potential, conducted within 3 days before the first dose and during safety follow-up.
- **b:** T3 is preferred; if not feasible, measure free T3.
- **c:** WBC differential includes lymphocytes, neutrophils, monocytes, basophils, and eosinophils.
- **d:** If urine protein is ≥2+, perform a 24-hour urine protein quantification.

Laboratory tests during the screening period must be completed within 7 days before the first dose of the study treatment. Exceptions include hepatitis and thyroid serology, which can be conducted within 28 days (close-label phase) or 21 days before the first dose. After Cycle 1, pre-treatment laboratory safety tests can be conducted within 3 days before dosing unless otherwise specified in the flowchart.

Laboratory test results must be reviewed and deemed acceptable by the investigator or qualified personnel before each study treatment administration, unless otherwise specified in the flowchart. Laboratory abnormalities related to drug-associated adverse events should be followed up until resolved. If laboratory test results are within normal ranges at the end of treatment, repeated tests are not required.

**7.2.2.2 Pregnancy Test**

All females considering participating in the trial who have not undergone surgical sterilization or are not menopausal must undergo a pregnancy test within 3 days before the first dose of the study drug. If the urine test is inconclusive, a serum test is required. Subjects with positive or borderline-positive results must be excluded or terminated from the study.

**7.2.2.3 Specimens for Central Laboratory Tests**

Biomarker testing (e.g., PD-L1, TMB) for this study will be conducted by the sponsor at designated laboratories. Specimen collection times, storage, and transportation instructions are detailed in the study procedure manual.

**7.3 Biomarker Analysis**

**7.3.1 Tissue Biomarkers**

Subjects meeting the inclusion criteria must provide qualified tumor tissue samples (tissue blocks or unstained slides) at baseline for PD-L1 immunohistochemistry and other analyses, based on investigator selection and ethical committee approval. Acceptable tissue samples include:

1. Archived tumor tissue meeting pathology quality control standards.
2. Newly collected tumor tissue during the screening period.

Unstained slides should be at least 5-15 slides with a thickness of 4-5 microns. Sampling requirements, sample storage, transportation, and analysis are detailed in the laboratory manual provided by the designated laboratory.

**7.3.2 Blood Biomarkers**

Subjects must provide a 10 ml whole blood sample at the following time points: before the first dose, at each efficacy evaluation during treatment, and before starting the next treatment, based on investigator selection and ethical committee approval. Biomarker research includes but is not limited to TCR sequencing analysis. Sampling methods, sample storage, transportation, and analysis are detailed in the laboratory manual.

**7.3.3 Storage and Disposal of Biological Samples**

Samples will be processed or destroyed with anonymization. Additional analyses may be conducted on anonymized, combined samples to further assess and validate analytical methods. Results from these analyses may be reported separately from the Clinical Study Report (CSR).

Sample reproducibility analysis, if performed, will be conducted concurrently with bioanalytical testing of trial samples. These results will be reported separately in a bioanalytical report.

**8 Safety Reporting and Adverse Event Management**

**8.1 Definition of Adverse Events**

An adverse event (AE) is defined as any unfavorable and unintended medical occurrence in a clinical trial subject, occurring from the time of signing the informed consent, regardless of its relationship to the study drug. AEs include but are not limited to:

- Worsening of pre-existing medical conditions/diseases (including symptoms, signs, laboratory abnormalities) existing before entering the clinical trial.
- New adverse medical conditions (including symptoms, signs, newly diagnosed diseases).
- Abnormal, clinically significant laboratory results.

**8.2 Definition of Serious Adverse Events**

A serious adverse event (SAE) is an AE that meets at least one of the following criteria:

- Results in death, excluding death due to disease progression of the study indication.
- Is life-threatening (defined as posing a risk of death when the AE occurs, not including events that might cause death if they worsen).
- Requires hospitalization or prolongs an existing hospitalization, excluding:
  - Rehabilitation facilities.
  - Nursing homes.
  - Routine emergency room admission.
  - Same-day surgeries (e.g., outpatient/day/non-hospitalized surgeries).
  - Hospitalization unrelated to AE exacerbation or prolongation itself is not an SAE (e.g., hospitalization for a pre-existing condition without a new AE or worsening of the condition).
- Results in permanent or significant disability/incapacity.
- Causes congenital abnormalities/birth defects.
- Other significant medical events: events that endanger the subject or require medical intervention to prevent any of the above.

**8.3 Evaluation of Adverse Events**

All AEs will be evaluated by the investigator using the NCI Common Terminology Criteria for Adverse Events (CTCAE) Version 5.0. Any change in CTCAE grade will be recorded on the AE case report form (CRF)/worksheet.

All AEs, regardless of CTCAE grade, must be assessed for seriousness.

**8.4 Recording Adverse Events**

The investigator should record AEs or SAEs using medical terminology/concepts, avoiding colloquialisms and abbreviations. All AEs (including SAEs) must be recorded on the AE section of the electronic CRF (eCRF).

**8.4.1 AE Collection Period** The investigator should learn about AEs by asking non-leading questions of the subject.

- All AEs, including SAEs, will be collected from the time of signing the informed consent until 30 days after the last administration, regardless of whether observed by the investigator or spontaneously reported by the subject.
- From 30 to 90 days post-last administration, the investigator must report all SAEs and AEs related to the study drug or study procedures.
- After 90 days post-last administration, the investigator should report any SAE considered related to the study drug or study procedures.
- If a new anti-tumor treatment is started within 90 days post-last administration, only SAEs related to the study drug need to be recorded.

**8.4.2 Follow-up of Adverse Events** AEs should be followed up until resolution to baseline or Grade 0-1, or until the investigator deems further follow-up unnecessary (e.g., recovery is not possible or has already improved). If an AE does not resolve, a reasonable explanation must be recorded in the CRF. Regardless of its relationship to the study drug, the resolution status and date of the AE or SAE should be recorded in the CRF and medical records.

**8.4.3 Contents of AE Records** Investigators should record any AE comprehensively, including diagnosis (if no diagnosis, record symptoms, signs including laboratory abnormalities), start and end dates and times (if applicable), CTCAE severity grade and changes (events of Grade 3 or higher), whether it is an SAE, actions taken regarding the study drug, treatment given for the AE, and the outcome. The relationship of the AE to the study drug should also be noted.

For SAEs, the investigator must also provide the date the AE met SAE criteria, the date the investigator became aware of the SAE, the basis for the SAE criteria, hospitalization dates, discharge dates, possible cause of death, date of death, whether an autopsy was performed, causality assessment with study procedures, causality assessment with other drugs, and other possible causes of the SAE. The investigator should provide the rationale for the causality assessment and a description of the SAE. The SAE description should include the subject's ID, age, gender, height, weight; study drug indication and disease stage; clinical course of the SAE; relevant laboratory test results (including test times, units, and normal ranges); relevant medical history, comorbid conditions, and their duration; relevant medication history, concomitant medications, including treatment start, duration, and dosage; and detailed information on the study drug treatment start, duration, and dosage.

**8.5 Rapid Reporting of SAEs and Pregnancy**

**SAE Reporting:** SAEs must be reported from the time of signing the informed consent until 90 days after the last administration. If an SAE occurs, the investigator must complete the Serious Adverse Event Report Form immediately and report it to the sponsor within 24 hours. It must also be reported to the national regulatory authority and ethics committee as required by Chinese regulations within 24 hours.

If an SAE outside the specified reporting period is deemed related to the study drug, it must also be reported.

**Pregnancy:** Given the potential for embryotoxicity with similar drugs, all subjects of childbearing potential must use effective contraception during the clinical trial.

If a female subject becomes pregnant during the clinical trial, she must be withdrawn from the study, and the pregnancy must be reported to the sponsor within 24 hours of becoming aware. The pregnancy report/follow-up form must be completed. If a male subject's partner becomes pregnant during the trial, the subject continues the trial, and the pregnancy must be reported within 24 hours.

The investigator should monitor and follow up on the pregnancy outcome until 8 weeks after delivery and report the outcome to the sponsor.

Pregnancy outcomes such as stillbirth, spontaneous abortion, fetal abnormalities (any congenital anomalies/birth defects), or medically indicated abortions are considered SAEs and must be reported as SAEs.

If an SAE occurs during pregnancy, it must be reported according to SAE reporting procedures.

**8.6 Immune-Related Adverse Events**

Due to the mechanism of action of sintilimab, which involves T-cell activation and proliferation, immune-related adverse events (irAEs) may be observed during the study. Subjects should be monitored for signs and symptoms of irAEs. If no clear alternative cause is identified (e.g., infection), it should be considered that the disease signs or symptoms occurring in the subject may be related to the immune system.

Dose adjustments and management principles for sintilimab in response to adverse events are detailed in Sections 5.3.2 and 5.3.3 of the protocol.

**9 Statistical Analysis**

**9.1 Sample Size Calculation**

In this single-arm, phase II trial, the hypothesis is that the combination therapy of sintilimab, anlotinib, and nab-paclitaxel will achieve an ORR of at least 50% in patients with relapsed ES-SCLC. Considering the historical ORR for topotecan, a standard second-line treatment, is around 25%, a 25% absolute improvement in ORR is deemed highly clinically meaningful for this combination therapy, thus setting the target ORR at 50%. With a target ORR of 50% for the combination therapy, enrolling 25 participants will provide approximately 80% power to demonstrate that the ORR rate of the combination therapy is greater than 25% (the historical rate for topotecan) at a significance level of one-sided 0.025. This means the lower bound of the 95% confidence interval (CI) should exclude 25%.

**9.2 Statistical Analysis Methods**

**9.2.1 General Statistical Methods**

Continuous variables will be described using median (minimum, maximum). Categorical variables will be described using frequency (percentage).

**9.2.2 Efficacy Analysis**

**9.2.2.1 Primary Endpoint Analysis**

**Progression-Free Survival (PFS)** PFS is defined as the time from the start of treatment to the first imaging-confirmed disease progression or death (whichever occurs first). Disease progression is determined by the investigator according to RECIST v1.1 criteria. Subjects who have not experienced disease progression or death at the time of analysis will be censored at the date of the last tumor assessment. The median PFS and its 95% CI will be estimated using the Kaplan-Meier method, and a survival curve will be plotted.

**9.2.2.2 Secondary Endpoint Analysis**

**Objective Response Rate (ORR)** ORR is defined as the proportion of subjects in the analysis population who achieve complete response (CR) or partial response (PR) as judged by the investigator according to RECIST 1.1 criteria. ORR = (number of subjects with CR + PR) / total number of subjects * 100%. The ORR and its 95% CI will be estimated using the binomial distribution, as well as the difference and its 95% CI. CR and PR determinations should be based on the best tumor response assessment during the study, confirmed twice.

**Disease Control Rate (DCR)** DCR is defined as the proportion of subjects who achieve CR, PR, or stable disease (SD). DCR = (number of subjects with CR + PR + SD) / total number of subjects * 100%. The DCR and its 95% CI will be estimated using the binomial distribution.

**Duration of Response (DOR)** DOR is defined as the interval from the first documentation of response to disease progression or death (whichever occurs first). The median DOR will be estimated using the Kaplan-Meier method, and a survival curve will be plotted.

**Overall Survival (OS)** OS is defined as the time from the start of treatment to death from any cause. A survival curve will be plotted.

**9.2.3 Safety Analysis**

The safety analysis set (SS) will be used for safety analysis. Safety indicators include adverse events, laboratory tests, vital signs, and ECG data.

**9.2.3.1 Adverse Event Analysis**

Adverse events (AEs) will be summarized for each treatment group, including adverse reactions, immune-related adverse events/reactions, serious adverse events/reactions, AEs leading to dose reduction and treatment interruption, AEs leading to study withdrawal, and AEs leading to death. The severity of AEs and adverse reactions will be graded according to NCI CTCAE version 5.0. All AEs and adverse reactions will also be summarized by system organ class (SOC) and preferred term (PT).

AEs will be summarized by treatment group, and overall AE summaries will be provided.

**9.2.3.2 Laboratory and Other Safety Data Analysis**

Laboratory tests, vital signs, ECGs, and physical examination data will be summarized by baseline data, post-treatment data, and changes from baseline at each visit and by treatment group. For categorical data, shift tables will be used to describe changes from baseline to each post-treatment visit in terms of normality and clinical significance.

**9.2.3.3 Drug Exposure**

Drug exposure during the study, including adherence to the study medication, treatment duration (number of cycles), dose adjustments, and cumulative dose adjustments during treatment will be summarized.

**9.2.4 Baseline Characteristics of Subjects**

Descriptive statistics will be used to summarize demographic characteristics (gender, age), diagnosis and treatment information for the indication (tumor type, pathological diagnosis, clinical stage, prior treatments), baseline tumor assessments (number, location, and total length of target and non-target lesions), and other baseline information such as height, weight (BMI, body surface area), vital signs, ECOG PS score, laboratory tests, prior/concomitant/new concomitant medications, etc.

**10 Quality Assurance and Quality Control**

According to GCP guidelines, the sponsor is responsible for implementing and maintaining a quality assurance and quality control system according to standard operating procedures to ensure that the clinical trial is conducted, and data is collected, recorded, and reported in compliance with the protocol, GCP, and applicable regulatory requirements.

**11 Ethics**

**11.1 Ethics Committee**

The investigator or their authorized representative will prepare the necessary documents to be submitted to the ethics committee (EC) of the research center, including the trial protocol, informed consent form (ICF), investigator's brochure, subject recruitment materials or advertisements, and other documents required by regulations. The EC approval must specify the protocol title, number, version number, and approval date. The research center must comply with the EC's requirements, which may include protocol amendments, ICF amendments, subject recruitment materials amendments, local safety reporting requirements, periodic reports and updates, and submission of the final report. All these documents and EC approvals must be provided to the sponsor or its designated personnel.

**11.2 Ethics of the Study**

The study process and informed consent should comply with the Declaration of Helsinki, relevant GCP requirements, and Chinese laws and regulations related to drug and data protection. GCP provides ethical and scientific quality standards for the design, conduct, recording, and reporting of clinical trials involving human subjects. This study will be conducted in accordance with GCP and applicable national regulations, adhering to the ethical principles of the Declaration of Helsinki to protect the rights, safety, and well-being of subjects. The investigator must follow the procedures specified in this protocol, and any protocol deviations will be reported to the EC or regulatory authorities.

**11.3 Subject Information and Informed Consent**

Before any study-related procedures begin, the potential risks and benefits of the study must be explained to potential subjects using an ICF in simple, easy-to-understand language. The ICF must clearly state that participation is voluntary, the potential risks and benefits of the study, and that subjects may withdraw from the study at any time. The investigator must obtain written consent from the subject or their legal representative after fully explaining the study details, satisfactorily answering all questions, and allowing sufficient time for consideration. All signed ICFs must be kept in the investigator's documentation or subject file. The investigator is responsible for explaining the content of the ICF to the subjects and obtaining their signed and dated ICF before starting the study. A copy of the signed ICF must be given to the subject. The informed consent process must be documented in the trial's source documents.

**11.4 Subject Data Protection**

The ICF will include information on data protection and privacy. Precautions will be taken to ensure the confidentiality of the documents to prevent identification of the subjects. In certain situations, specific personnel may access the subject's genetic data and personal identification codes, such as in medical emergencies where the investigator, their representative, or researcher has access to the subject's genetic data. Additionally, regulatory authorities may require access to the relevant documents.

**12 Study Management**

**12.1 Data Handling and Record Keeping**

Documents related to the clinical trial (protocol and amendments, completed CRFs, signed ICFs, etc.) must be stored and managed in accordance with GCP requirements. Research centers should retain these documents for 5 years after the study ends. Research documents should be stored reasonably to allow future access or data tracing. Safety and environmental risks should be considered when storing documents.

**12.2 Access to Original Data/Files**

The investigator agrees to provide direct access to all study-related documents, including subject records, to relevant regulatory authorities.

**12.3 Protocol Amendments**

Any appropriate amendments to the protocol during the study will be communicated and agreed upon with the investigator. All amendments should be kept as addendums to the protocol. Any modifications to the protocol must be submitted to the ethics committee for approval or filing, as required.

**12.4 Investigator Responsibilities**

The investigator will conduct this study following the protocol, the ethical principles of the Declaration of Helsinki, Chinese GCP, and relevant regulatory requirements. Detailed responsibilities of the investigator are listed in Chapter 5 (Investigator Responsibilities) of Chinese GCP (Order No. 3).

**12.5 Publication Policy**

All data generated from this study are considered confidential information of the investigator. The investigator has the right to publish the study results. Information on the investigator's publication policy will be described in the clinical trial agreement. All information related to this trial (not limited to the following documents: protocol, investigator brochure) must be kept strictly confidential. The investigator must recognize that the scientific or medical information obtained from this trial may have commercial value.

**12.6 Finance and Insurance**

The investigator will purchase insurance for subjects participating in this study according to local regulations and minimum requirements. The insurance-related terms will be kept in the study file.

**Referneces**

1. Bray F, Ferlay J, Soerjomataram I, et al. Global Cancer Statistics 2018: GLOBOCAN Estimates of Incidence and Mortality Worldwide for 36 Cancers in 185 Countries. CA: A Cancer Journal for Clinicians 2018; 0:1-31.

2. Govindan R, Page N, Morgensztern D, et al. Changing epidemiology of small-cell lung cancer in the United States over the last 30 years: analysis of the surveillance, epidemiologic, and end results database. J Clin Oncol. 2006 Oct 1;24(28):4539-44.

3. Toh CK, Gao F, Lim WT, et al. Differences between small-cell lung cancer and non-small-cell lung cancer among tobacco smokers. Lung Cancer. 2007 May;56(2):161-6.

4. Sun JM, Choi YL, Ji JH, et al. Small-cell lung cancer detection in never-smokers: clinical characteristics and multigene mutation profiling using targeted next-generation sequencing. Ann Oncol. 2015 Jan;26(1):161-6.

5. Sundstrom S, Bremnes RM, Kaasa S, et al. Cisplatin and Etoposide Regimen Is Superior to Cyclophosphamide, Epirubicin, and Vincristine Regimen in Small-Cell Lung Cancer: Results from a Randomized Phase III Trial With 5 Years' Follow-Up. [J]. Journal of Clinical Oncology,2002,24(24):4665-4672.

6. Siu LL, Shepherd FA, Murray N, et al. Influence of age on the treatment of limited-stage small-cell lung cancer. [J]. Journal of Clinical Oncology,1996,3(3):821-828.

7. CHENG Y, WANG Q M, LI K, et al. Anlotinib as third-line or further-line treatment in relapsed. SCLC: a multicentre, randomized, double-blind phase 2 trial. 2018 World Conference on Lung Can­cer. Abstract. OA13. 03.

8. CHENG Y, WANG QM, Li K, et al. Overall survival (OS) update in ALTER 1202: Anlotinib as third­line or further-line treatment in relapsed SCLC. European Society for Medical Oncology Congress 2019. Abstract. 17380.

9. CHENG Y, WANG Q M, LI K, et al. The impact of Anlotinib for relapsed SCLC patients with brain 1netastases: a subgroup analysis of ALTER 1202. 2019 World Conference on Lung Can­cer. Abstract. P2. 12-26.

10. McDermott DF, Atkins MB. PD-1 as a potential target in cancer therapy [J]. Cancer Med, 2013,2(5):662-673.

11. Barbee MS, Ogunniyi A, Horvat TZ, et al. Current status and future directions of the immune checkpoint inhibitors ipilimumab, pembrolizumab, and nivolumab in oncology [J]. Ann Pharmacother, 2015,49(8):907-937.

12. Horn L, Reck M, Spigel DR. The future of immunotherapy in the treatment of small cell lung cancer. Oncologist 2016;21:910-921.

13. Antonia SJ, Lopez-Martin JA, Bendell J, et al. Nivolumab alone and nivolumab plus ipilimumab in recurrent small-cell lung cancer (CheckMate 032): a multicentre, open-label, phase 1/2 trial. Lancet Oncol 2016;17:883-895.

14. Ready NE, Ott PA, Hellmann MD, et al. Nivolumab Monotherapy and Nivolumab Plus Ipilimumab in Recurrent Small Cell Lung Cancer: Results From the CheckMate 032 Randomized Cohort. J Thorac Oncol. 2019 Oct 17. pii: S1556-0864(19)33531-2.

15. Chung HC, Piha-Paul SA, Lopez-Martin J, et al. CT073 - Pembrolizumab after two or more lines of prior therapy in patients with advanced small-cell lung cancer (SCLC): Results from the KEYNOTE-028 and KEYNOTE-158 studies [abstract]. AACR Annual Meeting. Atlanta, GA; 2019:Abstract CT073.

16. Ott PA, Elez E, Hiret S, et al. Pembrolizumab in patients with extensive-stage small-cell lung cancer: results from the phase Ib KEYNOTE-028 study. J Clin Oncol 2017;35:3823-3829.

17. Reck M, Vicente D, Ciuleanu T, et al. LBA5: Efficacy and safety of nivolumab (nivo) monotherapy versus chemotherapy (chemo) in recurrent small cell lung cancer (SCLC): Results from CheckMate 331 [abstract].Ann Oncol 2018;29:43.

18. Liu S, Mansfield A, Szczesna S, et al. First-Line Atezolizumab plus Chemotherapy in Extensive-Stage Small-Cell Lung Cancer. NEJM. 2018;doi:10.1056/NEJMoa1809064.

19. L. Paz-Ares, Y. Chen, N. Reinmuth, et al. Overall survival with durvalumab plus etoposide-platinum in first-line extensive-stage SCLC: results from the CASPIAN study. Presented at: IASLC 20th World Conference on Lung Cancer; September 7-10, 2019; Barcelona, Spain. Abstract OA02.02.

20. Wang S. abstr 1443. CSCO, 2018.

21. J Clin Oncol 37, 2019 (suppl; abstr e20546).

22. Jian WU ea. CSCO, abstr 1262 [J]. 2018.

**Appendix 1: Performance Status Score Standard (ECOG PS)**

| **Activity Score** | **Description** |
| --- | --- |
| 0 | Asymptomatic, fully active, able to carry on all pre-disease performance without restriction. |
| 1 | Symptomatic but completely ambulatory, restricted in physically strenuous activity but ambulatory and able to carry out work of a light or sedentary nature, e.g., light housework, office work. |
| 2 | Ambulatory and capable of all self-care but unable to carry out any work activities. Up and about more than 50% of waking hours. |
| 3 | Capable of only limited self-care, confined to bed or chair more than 50% of waking hours. |
| 4 | Completely disabled. Cannot carry on any self-care. Totally confined to bed or chair. |
| 5 | Dead. |

**Appendix 2: Calculations for Creatinine Clearance and Body Surface Area**

1. **Cockcroft-Gault Formula for Calculating Creatinine Clearance**

1.1 Formula for serum creatinine concentration in (mg/dL):

Male Creatinine Clearance (mL/min) = (140−age) × (weight in kg)72×Serum Creatinine (mg/dL)72×Serum Creatinine (mg/dL) (140−age) × (weight in kg)​

Female Creatinine Clearance (mL/min) = 0.85×(140−age) × (weight in kg)72×Serum Creatinine (mg/dL)0.85×72×Serum Creatinine (mg/dL) (140−age) × (weight in kg)​

1.2 Formula for serum creatinine concentration in (μmol/L):

Male Creatinine Clearance (mL/min) = (140−age) × (weight in kg)81×Serum Creatinine (μmol/L)81×Serum Creatinine (μmol/L) (140−age) × (weight in kg)​

Female Creatinine Clearance (mL/min) = 0.85×(140−age) × (weight in kg)81×Serum Creatinine (μmol/L)0.85×81×Serum Creatinine (μmol/L) (140−age) × (weight in kg)​

Note: Age is in years, weight is in kg.

1. **Body Surface Area (Stevenson Formula)**:

Body Surface Area (m²) = 0.00616×Height (cm)+0.01286×Weight (kg)−0.15290.00616×Height (cm)+0.01286×Weight (kg)−0.1529
